# Gibberellin Acts through Jasmonate to Control the Expression of *MYB21*, *MYB24*, and *MYB57* to Promote Stamen Filament Growth in *Arabidopsis*

**DOI:** 10.1371/journal.pgen.1000440

**Published:** 2009-03-27

**Authors:** Hui Cheng, Susheng Song, Langtao Xiao, Hui Meng Soo, Zhiwei Cheng, Daoxin Xie, Jinrong Peng

**Affiliations:** 1 Institute of Molecular and Cell Biology, Agency for Science, Technology and Research (A*STAR), Proteos, Singapore; 2 MOE Key Laboratory of Bioinformatics, Department of Biological Sciences and Biotechnology, Tsinghua University, Beijing, People's Republic of China; 3 Huanan Provincial Key Laboratory of Phytohormones and Growth Development, College of Bioscience and Biotechnology, Hunan Agricultural University, Changsha, People's Republic of China; 4 College of Animal Sciences, Zhejiang University, Hangzhou, People's Republic of China; The University of North Carolina at Chapel Hill, United States of America

## Abstract

Precise coordination between stamen and pistil development is essential to make a fertile flower. Mutations impairing stamen filament elongation, pollen maturation, or anther dehiscence will cause male sterility. Deficiency in plant hormone gibberellin (GA) causes male sterility due to accumulation of DELLA proteins, and GA triggers DELLA degradation to promote stamen development. Deficiency in plant hormone jasmonate (JA) also causes male sterility. However, little is known about the relationship between GA and JA in controlling stamen development. Here, we show that *MYB21*, *MYB24*, and *MYB57* are GA-dependent stamen-enriched genes. Loss-of-function of two DELLAs *RGA* and *RGL2* restores the expression of these three *MYB* genes together with restoration of stamen filament growth in GA-deficient plants. Genetic analysis showed that the *myb21-t1 myb24-t1 myb57-t1* triple mutant confers a short stamen phenotype leading to male sterility. Further genetic and molecular studies demonstrate that GA suppresses DELLAs to mobilize the expression of the key JA biosynthesis gene *DAD1*, and this is consistent with the observation that the JA content in the young flower buds of the GA-deficient quadruple mutant *ga1-3 gai-t6 rga-t2 rgl1-1* is much lower than that in the WT. We conclude that GA promotes JA biosynthesis to control the expression of *MYB21*, *MYB24*, and *MYB57*. Therefore, we have established a hierarchical relationship between GA and JA in that modulation of JA pathway by GA is one of the prerequisites for GA to regulate the normal stamen development in *Arabidopsis*.

## Author Summary

Gibberellin and jasmonate are plant hormones that mediate diverse plant developmental processes and responses to the environment. Deficiency in either gibberellin or jasmonate causes male sterility, in part due to the short stamen filament conferred. In this report, we sought to study the interaction between gibberellin and jasmonate during stamen filament development. We focused on three *MYB* genes, namely *MYB21*, *MYB24*, and *MYB57*, which have been proven to be essential for stamen filament development in *Arabidopsis*. These three *MYB* genes are regulated by both gibberellin and jasmonate. We performed various molecular analyses and found that GA activates the expression of *DAD1* and *LOX1*, two genes essential for jasmonate biosynthesis. The hypothesis of GA regulating JA biosynthesis is proved by the fact that the JA content in the young flower buds of the *ga1-3 gai-t6 rga-t2 rgl1-1* quadruple mutant is much lower than that in the WT. This evidence demonstrates that GA promotes the production of jasmonate and high level of jasmonate will induce the expression of *MYB21*, *MYB24*, and *MYB57* to promote stamen filament development. This is most likely the first molecular and genetic evidence to show how gibberellin and jasmonate interact to control stamen filament development.

## Introduction

*Arabidopsis* flowers are organized into four concentric whorls of distinct organs (sepals, petals, stamens and pistils). Stamens, the male reproductive organs of flowering plants, form the third whorl. Processes of stamen filament elongation and anthesis are precisely controlled so that they coincide with the pistil development to determine the fertility [1]. Mutations that impair stamen development such as filament elongation, pollen maturation or anther dehiscence will result in male sterility [2],[3]. Many genes have been found to control stamen development [4],[5]. Stamen development is also subjected to hormonal control. For example, mutations affecting biosynthesis of two plant hormones gibberellin (GA) (e.g *ga1-3* mutation) and jasmonate (JA) (e.g *opr3* mutation) both confer male sterile phenotype due to failure of stamen filament elongation and of completion of anthesis and anther dehiscence [6],[7].

A severe *Arabidopsis* GA-deficient mutant, *ga1-3* exhibits retarded growth at both vegetative and reproductive stages [7]. The development of floral organs, especially petals and stamens, is impaired in the *ga1-3* mutant. Detailed anatomical analysis showed that the male sterile phenotype of *ga1-3* is due to the arrestment of stamen filament cell elongation and failure of completion of anthesis [8]. Application of exogenous GA can restore all the floral defects of *ga1-3* [7]. Further studies revealed that the arrested floral development in *ga1-3* is mediated by DELLA proteins [8],[9]. DELLAs are a subfamily of the plant GRAS family of putative transcription regulators [10],[11] and have been revealed to function as negative regulators of GA response in diverse plant species including *Arabidopsis*, barley, rice and wheat etc [12]–[17]. There are five DELLAs in *Arabidopsis*, namely GAI, RGA, RGL1, RGL2 and RGL3 [18],[19]. Genetic studies have shown that RGA, RGL2 and RGL1 act synergistically in repressing petal and stamen development and GA triggers the degradation of these DELLAs to promote floral development [8], [9], [20]–[22]. Severe JA deficient mutant *opr3* and JA-signaling mutant *coi1* also displayed retarded filament elongation, delayed anther dehiscence, and reduced pollen viability. As a consequence, the *opr3* and *coi1* mutants are male sterile [6],[23]. Application of exogenous JA can fully restore the stamen development to *opr3* [6].

It is intriguing to know whether GA-mediated and JA-mediated stamen development are via two parallel pathways or in a hierarchical way to control stamen development. In *Arabidopsis*, the known GA-response genes encoding transcription factors involved in stamen development are *GAMYBs* (*MYB33* and *MYB65*), a subset of *MYB* genes [24]. GAMYB is the best characterized GA-regulated transcription factor and was first identified in barley. GAMYB was found to bind to the GA-response elements (GARE) in the promoter of the *α–amylase* gene in cereals [25],[26]. Genetic studies showed that *Arabidopsis GAMYBs* (*MYB33* and *MYB65*) are essential to anther maturation but not for the elongation of stamen filament in *Arabidopsis* [24]. Previous studies have shown that GA regulates *GAMYB* through DELLA protein SLN1 and SLR1 in barley and rice, respectively [27],[28]. However, several reports failed to identify *MYB33* and *MYB65* as GA-inducible genes in *Arabidopsis* and these two *MYB* genes are in fact regulated at the post-transcriptional level by miRNA159 [24], [29]–[31]. Two recent reports showed that three *MYB* genes (*MYB21*, *MYB24* and *MYB108*) are responsive to JA treatment in *opr3* mutant and loss-of-function of *MYB21* and *MYB24* resulted in a short stamen phenotype [32] whereas *MYB108* is involved in stamen and pollen maturation but not stamen filament elongation [33]. Interestingly, in an expression profiling study, we identified several *MYBs* including *MYB21*, *MYB24*, and *MYB57* as DELLA-downregulated genes in *ga1-3* flower buds [30]. This fact prompted us to investigate if there might be a cross-talk between GA signaling and JA signaling during stamen development.

*MYB21* and *MYB24* have been shown to be expressed in all four whorls of the flower [32],[34],[35]. In this report, we showed that *MYB21*, *MYB24*, and *MYB57* are down-regulated in the *ga1-3* single mutant and the sterile quadruple mutant *ga1-3 gai-t6 rga-t2 rgl1-1* (loss-of-function of *GAI*, *RGA*, *RGL1* three DELLA genes but *RGL2* is normal) but restored to wild type levels in the fertile penta mutant *ga1-3 gai-t6 rga-t2 rgl1-1 rgl2-1* (loss-of-function of GAI, RGA, RGL1 and RGL2 four DELLA genes). We also showed that absence of the four DELLAs (GAI, RGA, RGL1 and RGL2) cannot suppress the short stamen phenotype conferred by the loss-of-function of *MYB21* and *MYB24*. In addition, we observed that application of exogenous JA onto the *ga1-3 gai-t6 rgl1-1 rgl2-1* quadruple mutant flower buds could restore the expression of *MYB21*, *MYB24* and *MYB57* whereas application of exogenous GA onto *opr3* mutant flower buds failed to increase the expression of these three *MYBs*. Most importantly, we showed that GA upregulates JA-biosynthetic genes *DAD1* and *LOX1* and the JA content in the young flower buds of the GA-deficient quadruple mutant *ga1-3 gai-t6 rga-t2 rgl1-1* is much lower than that in the WT and penta mutant. Therefore, we conclude that GA upregulates the *DAD1* and *LOX1* expression to promote JA production to promote the expression of the three *MYBs* necessary for stamen filament development.

## Results

### Identification of DELLA-Repressed Stamen-Enriched Genes

**Figure 1:**
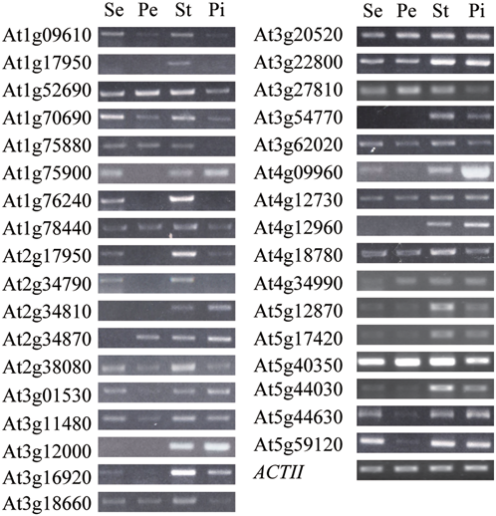
Identification of DELLA-Repressed Stamen-Enriched Genes. At least three independent samples were used for RT-PCR analysis for each individual gene and a representative gel picture for each gene was shown here. Total 34 genes were identified as DELLA-repressed stamen-enriched genes (summarized in Table 1) based on their relative more abundant expression in the stamen than in one or more of the rest of the floral organs. Primer pairs corresponding to these genes were listed in Table S1. Se, sepal; Pe, petal; St, stamen; Pi, pistil.

The *ga1-3* mutant is retarded in floral development, suggesting that the transcriptome for floral development in the *ga1-3* mutant must be kept at a repressive state. Conversely, the fact that the *ga1-3 gai-t6 rga-t2 rgl1-1 rgl2-1* mutant (penta mutant) confers GA independent flowering suggests that the transcriptome responsible for floral development must have been constitutively activated in the penta mutant. We compared the expression profiles between *ga1-3* and *ga1-3 gai-t6 rga-t2 rgl1-1 rgl2-1* and identified 360 DELLA-repressed and 273 DELLA-activated genes essential for floral development [30]. To identify DELLA-repressed stamen-enriched genes, we examined expression of 43 DELLA-repressed genes in the sepal, petal, stamen and pistil via semi-quantitative RT-PCR. These 43 genes were chosen based on two criteria: 1) they are homologous to transcription factors known to regulate GA-response (e.g *MYB* gene family) and 2) genes whose expression showed drastic changes between the *ga1-3* and penta mutant [30]. Only genes whose expression are either enriched in the stamen or highly expressed in the stamen and also in some other floral organs but not ubiquitously highly expressed in all four floral organs were classified as the stamen-enriched genes. A total of 34 genes, including two *APG-like* genes (At1g75880, At1g75900) and three genes (*IRX1*, *IRX3*, *IRX5*) encoding the cellulose synthase subunits which are known to be enriched in the stamen, were identified as DELLA-repressed stamen-enriched genes (Figure 1; Table 1).

**Table 1:** RT-PCR examination of DELLA-down genes in different floral organs.

Gene ID	Gene description	DELLA-Down
At1g09610	Hypothetical protein	confirmed
At1g17950	MYB52	confirmed
At1g52690	Late embryogenesis abundant protein	confirmed
At1g70690	Unknown	confirmed
At1g75880	APG-like	confirmed
At1g75900	APG-like	confirmed
At1g76240	Hypothetical protein	confirmed
At1g78440	Gibberellin 2-oxidase	confirmed
At2g17950	Homeodomain transcription factor	confirmed
At2g34790	Berberrine bridge enzyme	confirmed
At2g34810	Berberrine bridge enzyme	confirmed
At2g34870	Unknown	confirmed
At2g38080	Putative diphenol oxidase	confirmed
At3g01530	MYB57	confirmed
At3g11480	Hypothetical	confirmed
At3g12000	S-locus related	confirmed
At3g15270	Squamose promoter binding 5	confirmed
At3g16920	Chitinase(GHF19)	confirmed
At3g18660	Hypothetical protein	confirmed
At3g20520	Hypothetical protein	confirmed
At3g22800	Extensin-like	confirmed
At3g27810	MYB21	confirmed
At3g54770	RNA binding protein	confirmed
At3g62020	Germin-like protein	confirmed
At4g12730	Putative pollen surface protien	confirmed
At4g12960	Unknown	confirmed
At4g18780	Cellulose synthase (IRX1)	confirmed
At4g34990	MYB32	confirmed
At5g12870	MYB46	confirmed
At5g17420	Cellulose synthase (IRX3)	confirmed
At5g40350	MYB24	confirmed
At5g44030	Cellulose synthase (IRX5)	confirmed
At5g44630	Terpene synthase	confirmed
At5g59120	Subtilisin-like serine protease	confirmed

Se: sepal, Pe: petal, St: stamen, Pi: pistil. Expression levels in different floral organs were based on the semi-quantitative RT-PCR results. “−” not detected, “+” faintly detected, “++” detected, “+++” strongly detected. DELLA-D: down-regulated by DELLA proteins.

### DELLAs Repress the Expression of *MYB21*, *MYB24*, and *MYB57*

**Figure 2:**
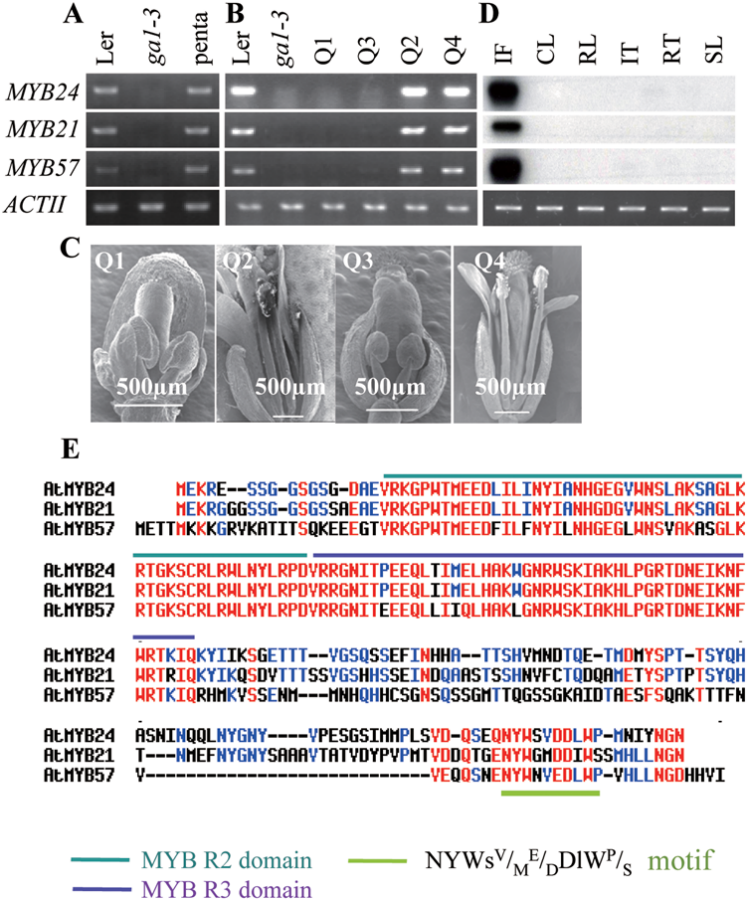
*MYB21*, *MYB24I*, and *MYB57* Are RGA- and RGL2-Repressible Floral Specific Genes. (A) RT-PCR analysis shows that the expression of the three *MYB* genes in the young flower buds are greatly reduced in *ga1-3* but restored to the WT level in *ga1-3 gai-t6 rgat2 rgl1-1 rgl2-1* (penta). (B–C) RT-PCR analysis shows that the repressed expression of the three *MYB* genes in *ga1-3* was restored in *ga1-3 gai-t6 rga-t2 rgl2-1* (Q2) and *ga1-3 rga-t2 rgl1-1 rgl2-1* (Q4) two quadruple mutants but not in *ga1-3 gai-t6 rgl1-1 rgl2-1* (Q1) and *ga1-3 gai-t6 rga-t2 rgl1-1* (Q3) two quadruple mutants (B). This restoration of *MYB* expression nicely correlates with the recovery of fertility in Q2 and Q4 (C). Total RNA used in RT-PCR analysis was extracted from the young flower buds. (D) RT-PCR analysis shows that the three *MYB* genes are floral specific genes. IF, inflorescence; CL, cauline leaves; RL, rosette leaves; IT, internodes; RT, roots; SL, siliques. (E) Amino acid alignment of MYB21, MYB24 and MYB57 proteins. The conserved R2 and R3 domains and the NYWSV/ME/DDlWP/S motif are highlighted in red, blue and green, respectively.

Three *MYB* genes, namely *MYB21*, *MYB24* and *MYB57*, were among the identified DELLA-repressed stamen-enriched genes (Figure 1; Table 1). Based on the phylogenetic tree, MYB24 and MYB21 are classified into the subgroup 19 of R2R3-MYB family [36]. MYB57 shares high similarity with this subfamily and is a close member to this subfamily [37]. Overall, MYB21 shares 61.6% and 51.0% identity with MYB24 and MYB57 at the amino acid level, respectively (Figure S1). The expression of these three *MYBs* in the young flower buds were reduced to a very low level in *ga1-3* but restored to the wild type (WT) level in the *ga1-3 gai-t6 rga-t1 rgl1-1 rgl2-1* penta mutant (Figure 2A). In order to find out which DELLA (RGL1, RGL2, RGA and GAI) is more effective in repressing the expression of *MYB21*, *MYB24* and *MYB57*, transcript levels of each individual *MYB* gene were studied in four quadruple mutants in which only one of the four *DELLA* genes remains intact. All three *MYB* genes were almost undetectable in the Q1 (*ga1-3 gai-t6 rgl1-1 rgl2-1*, wild type for *RGA*) and barely detectable in the Q3 (*ga1-3 gai-t6 rgl1-1 rga-t2*, wild type for *RGL2*) mutants but were detected at high levels in the Q2 (*ga1-3 rga-t2 rgl1-1 rgl2-1*, wild type for *GAI*) and Q4 (*ga1-3 gai-t6 rga-t2 rgl2-1*, wild type for *RGL1*) mutants (Figure 2B), suggesting that RGA and RGL2, but not GAI nor RGL1, were the more effective DELLAs in repressing the expression of these three *MYB* genes. Interestingly, we showed previously that while Q1 and Q3 mutants, as the *ga1-3* mutant, were retarded in floral development both Q2 and Q4 mutants produced normal fertile flowers (Figure 2C) [8]. Therefore, it seems there is a nice correlation between normal floral development and the expression of *MYB21*, *MYB24* and *MYB57*, suggesting that these three *MYBs* are probably necessary for normal floral development.

### *MYB21*, *MYB24*, and *MYB57* Function Redundantly in Controlling Stamen Filament Elongation

Expression analysis showed that *MYB21* and *MYB24* [32],[34] as well as *MYB57* are flower-specific genes (Figure 2D). To determine if the spatial and temporal expression patterns of *MYB21* and *MYB24* correlate with their proposed role during stamen filament elongation, we examined *MYB21* expression via *in situ* hybridization and generated *pMYB24:GUS* transgenic for examining *MYB24* expression. Our *in situ* hybridization result showed that, starting from floral stage 12 [1],[38], *MYB21* is expressed in the anther vascular tissue and in cells at the junction between anther and stamen filament (Figure S2A,B) where rapid filament elongation is hypothesized to occur starting from the floral stage 13 after a successful pollination [1]. *MYB21* expression is also detected in the nectaries and ovules (Figure S2A,B). Similarly, staining the young inflorescence of the *pMYB24::GUS* plants revealed that strong GUS activity was detected in the vascular tissue of stamen filament and sepals whereas only weak GUS activity was detectable in the petals starting from floral stage 12 (Figure S3A–E). GUS activity was also detected in the upper part of the pistils (Figure S3A–3E).

**Figure 3:**
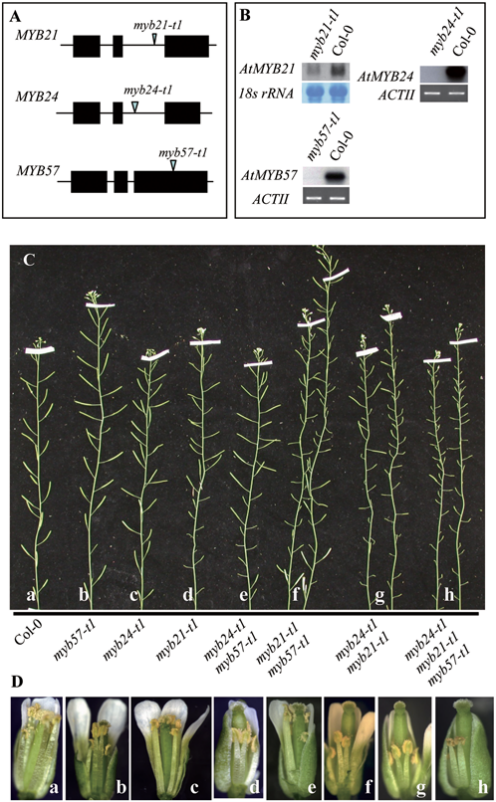
*MYB21*, *MYB24*, and *MYB57* Function Redundantly in Regulating the Stamen Filament Development. (A) Schematic diagram shows the respective T-DNA insertions in the three *MYB* genes. Black box: exon; black line: intron; triangle: T-DNA insertion site. (B) RT-PCR analysis of *MYB24* transcripts in *myb24-t1* and *MYB57* transcripts in *myb57-t1* and northern analysis of *MYB21* transcripts in *myb21-t1*. Total RNA for RT-PCR and northern analysis was extracted from the young flower buds. (C) Comparison of main shoots bearing siliques among different mutant lines as indicated. (D) Comparison of the stamen phenotype among different mutant lines as indicated. Genotypes for flowers a-h in (D) corresponds to that showed in (C).

To investigate their roles in GA-mediated floral organ development, we identified T-DNA insertional mutant lines corresponding to these three *MYB* genes from the Salk Institute Genomic Arabidopsis Laboratory (SIGnAL) database. Mutant alleles were confirmed (data not shown) and designated as *myb21-t1* (SALK_042711) for *MYB21*, *myb24-t1* (SALK_017221) for *MYB24*, and *myb57-t1* (SALK_065776) for *MYB57* (Figure 3A). *myb24-t1* and *myb57-t1* are both likely null alleles since *MYB24* and *MYB57* transcripts were undetectable in *myb24-t1* and *myb57-t1* mutant flower buds, respectively (Figure 3B). On the other hand, *MYB21* transcripts were still detectable in *myb21-t1* although its level was greatly reduced in the mutant, suggesting that *myb21-t1* is likely a leaky allele (Figure 3B). After two rounds of backcross, we found that *myb24-t1* and *myb57-t1* mutant plants were phenotypically indistinguishable from the WT control plant (Figure 3C; Table 2). However, in *myb21-t1* the early developed flowers (∼the first 10 flowers) bore short stamens (Figure S4) with greatly reduced fertility and only the late developed flowers yielded proper seed settings (Figure 3C; Table 2). A close look at the matured early flowers in *myb21-t1* showed that the stamens did produce pollens (Figure 3D, panel d). Cross-pollinating the pollens onto the *myb21-t1* stigma yielded seeds that were homozygous for *myb21-t1* and onto the WT stigma yielded *myb21-t1* heterozygous seeds (data not shown), demonstrating that the short stamen is responsible for the partial sterile phenotype. Although *myb21-t1* is likely a leaky allele, the short stamen phenotype conferred by the *myb21-t1* mutation is identical to a *MYB21* null allele we obtained later from Gabi-Kat stock (stock number N311167, data not shown).

**Table 2:** Seed settings in different mutants grown under LD condition.

Col-*0*/mutant	Number of Siliques	Number of Siliques With Seeds	Percentage of Siliques With Seeds
Col-0	21.1±5.1	20.7±5.0	98.1±2.7
*myb57*	22.3±5.7	20.3±6.6	89.4±1.27
*myb24*	28.1±3.8	27.3±3.8	97.3±2.9
*myb21*	26.3±4.8	17.1±5.4	64.2±10.7
*myb24myb57*	26.3±6.9	22.6±6.8	85.1±6.3
*myb21myb57*	30.6±6.4	9.4±6.5	29.5±17.3
*myb21myb24*	30.8±9.5	5.6±4.3	16.9±11.8
*myb21myb24myb57*	33.4±7.5	1.6±1.5	4.1±3.6

a Siliques formed in primary inflorescence of plants were scored at 45 days.

The WT-like phenotype displayed by *myb24-t1* and *myb57-t1* and mild floral phenotype displayed by *myb21-t1* suggest that these *MYB* genes might function redundantly during stamen development. To prove this hypothesis, crosses were made among homozygous *myb24-t1*, *myb21-t1* and *myb57-t1* plants. Three double mutants (*myb21-t1 myb24-t1*, *myb21-t1 myb57-t1*, and *myb24-t1 myb57-t1*) and one triple mutant (*myb21-t1 myb24-t1 myb57-t1*) were generated and used in our phenotypic analysis.

The flower development of *myb24-t1 myb57-t1* double mutant at all stages was indistinguishable from the WT control (Figure 3D; Table 2) [1]. Stamens in mature flowers of the *myb21-t1 myb24-t1* double mutant were shorter than that of the *myb21-t1* single mutant and shorter stamens were also observed in majority of the late developed mature flowers in the double mutant (Figure S4). As a result, the *myb21-t1 myb24-t1* double mutant is more severely sterile than *myb21-t1* by having fewer siliques with seed settings (Figure 3C and 3D; Table 2), an observation also reported by Mandaokar et al [32]. The *myb21-t1 myb57-t1* double mutant had shorter stamens in early developed mature flowers and some of the later flowers (Figure 3D; Figure S4) and its seed settings displayed an intermediate phenotype between *myb21-t1* single and *myb21-t1 myb24-t1* double mutants (Figure 3C and 3D; Table 2). Interestingly, cross-pollination showed that the short stamens in both *myb24-t1 myb21-t1* and *myb21-t1 myb57-t1* double mutant plants produced viable pollens (data not shown), suggesting that the short stamen (Figure S4) is responsible for the reduced fertility in these mutants.

The *myb21-t1 myb24-t1 myb57-t1* triple mutant, as the *myb21-t1 myb24-t1* double mutant, had short stamens but was even more severely sterile than *myb21-t1 myb24-t1* (Figure 3C and 3D; Figure S4; Table 2). For *myb21-t1 myb24-t1* double and *myb21-t1 myb24-t1 myb57-t1* triple mutant plants, we occasionally observed that while, in the same inflorescence, most of the flowers did not set or set very few seeds, some were able to develop normal siliques filled up with seeds (Figure 3C and 3D). Cross pollination showed the pollens produced by *myb21-t1 myb24-t1 myb57-t1* triple mutant plants were partial viable (data not shown), suggesting the short stamens in the triple mutants are the main cause of the sterility. It is possible that environmental factors (e.g. temperature) may influence male fertility in these mutants, an observation also reported for *MYB33* and *MYB65* [24]. Therefore, *MYB21*, *MYB24* and *MYB57* function redundantly to control the stamen filament development in the late developed flowers.

Since *MYB21* and *MYB24* are also expressed in sepals and petals, we examined the sepal and petal development in the single, double and triple *myb* mutants. As shown in Figure 3D, sepal development appeared normal in all mutants whereas petal development varied in different mutants. Petals in the *myb24-t1* and *myb57-t1* two single mutants grew to a final length longer than the pistils, as that did the WT petals. Petals in the *myb21-t1* single, *myb24-t1 myb57-t1* and *myb21-t1 myb57-t1* two double mutants grew to a final height parallel to the pistil (Figure 3D). Petals in the *myb21-t1 myb24-t1* double mutant grew just out of the sepals but ended at a lower level than the stigma (Figure 3D). The growth of petals in the *myb21-t1 myb24-t1 myb57-t1* triple mutant was arrested and the petals never grew out of the sepals (Figure 3D).

### *myb21-t1 myb24-t1* Is Epistatic to *gai-t6 rga-t2 rgl1-1 rgl2-1* in Controlling Stamen Filament Elongation

**Figure 4:**
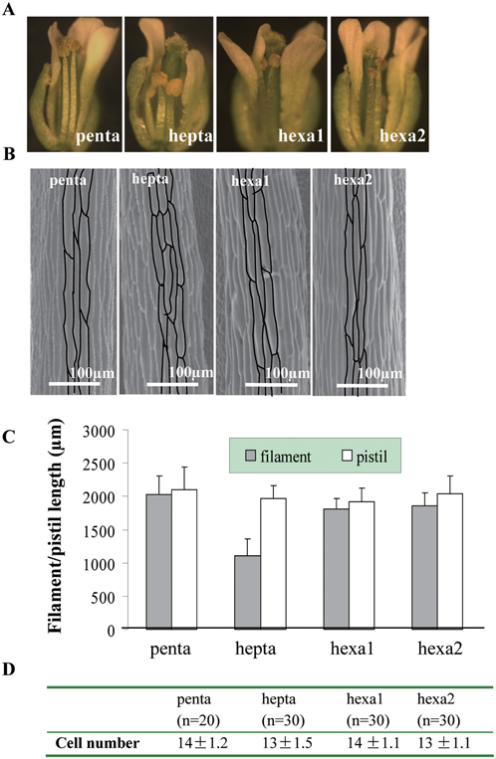
*myb21-t1 myb24-t1* Is Epistatic to *ga1-3 gai-t6 rga-t2 rgl1-1 rgl2-1* in Controlling Stamen Filament Elongation. (A) Comparison of the stamen phenotype among *ga1-3 gai-t6 rga-t2 rgl1-1 rgl2-1*(penta), *ga1-3 gai-t6 rga-t2 rgl1-1 rgl2-1 myb21-t1 myb24-t1* (hepta), *ga1-3 gai-t6 rga-t2 rgl1-1 rgl2-1 myb21-t1* (hexa1) and *ga1-3 gai-t6 rga-t2 rgl1-1 rgl2-1 myb24-t1* (hexa2). (B) SEM of stamen filament epidermal cells in the penta, hepta, hexa1 and hexa2 mutants. Segments shown were all from the middle part of the filament. Some individual cells were outlined with black lines for easy visualization. (C) Comparison of stamen and pistil lengths among different genotypes. Filament and pistil lengths were measured from SEM pictures (n = 30). (D) Average number of epidermal cells per stamen filament in penta, hepta, hexa1 and hexa2. n: number of stamens used in counting.

*MYB21* and *MYB24* were repressed in *ga1-3* but their expressions were restored to the WT level in the *ga1-3 gai-t6 rga-t2 rgl1-1 rgl2-1* penta mutant, suggesting that GA regulates *MYB21* and *MYB24* through inactivating DELLA proteins. Application of exogenous GA could not rescue the stamen development in *myb21 myb24* mutant (data not shown), suggesting that *MYB21* and *MYB24* are needed in GA-mediated stamen development. To further confirm this hypothesis, we crossed *myb21 myb24* with *ga1-3 gai-t6 rga-t2 rgl1-1 rgl2-1* to generate two hexa mutants (hexa1: *ga1-3 gai-t6 rga-t2 rgl1-1 rgl2-1 myb21-t1*; hexa2: *ga1-3 gai-t6 rga-t2 rgl1-1 rgl2-1 myb24-t1*) and one hepta mutant (*ga1-3 gai-t6 rga-t2 rgl1-1 rgl2-1 myb21-t1 myb24-t1*). The two hexa mutants overall appeared similar to each other and had wildtype-like stamens and were largely fertile (Figure 4A; Figure S5). The hepta mutant plant displayed no difference from the penta mutant plant in its vegetative growth. However, its mature flowers showed a short filament phenotype identical to that in the *myb21-t1 myb24-t1* double mutant (Figure 4A; Figure S5). This observation demonstrated that *myb21-t1 myb24-t1* double mutations are epistatic to DELLA mutations. SEM analysis showed that the short stamen phenotype in the hepta mutant was due to reduced cell length (Figure 4B and 4C), rather than to a reduction in cell number (Figure 4D). Therefore, *MYB21* and *MYB24* act downstream of DELLAs in GA signaling pathway to control the stamen filament development.

### GA Application Fails to Induce the Expression of *MYB21*, *MYB24* and *MYB57* in JA-Deficient Mutant

**Figure 5:**
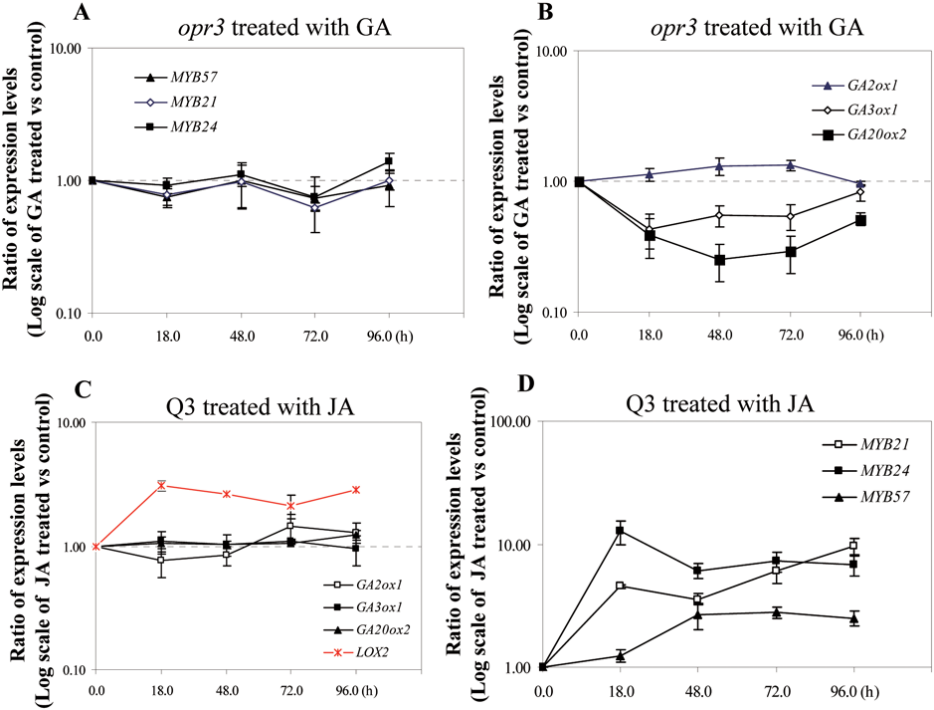
JA-Deficiency Specifically Blocks GA-Signaling Leading to the Induction of Expression of *MYB21*, *MYB24*, and *MYB57*. (A–B) Semi-quantitative analysis of *MYB21*, *MYB24*, *MYB57* (A), *GA2ox1*, *GA3ox1* and *GA20ox2* (B) expression in the *opr3* mutant flowers at 18, 48, 72 and 96 hrs after GA treatment. Data were averaged from 2–4 batches of independently treated samples and *ACTII* was used as the normalization control. The graph was drawn based on Log_10_ scale of the ratio of the expression levels of GA treated versus untreated samples. (C–D) Semi-quantitative analysis of *LOX2* (in red line), *GA2ox1*, *GA3ox1* and *GA20ox2* (C), *MYB21*, *MYB24* and *MYB57* (D) expression in the *ga1-3 gai-t6 rga-t2 rgl1-1* (Q3) mutant flowers at 18, 48, 72 and 96 hrs after JA treatment. Data were averaged from 2–4 batches of independently treated samples and *ACTII* was used as the normalization control. The graph was drawn based on Log_10_ scale of the ratio of the expression levels of JA treated versus untreated samples.

**Figure 6:**
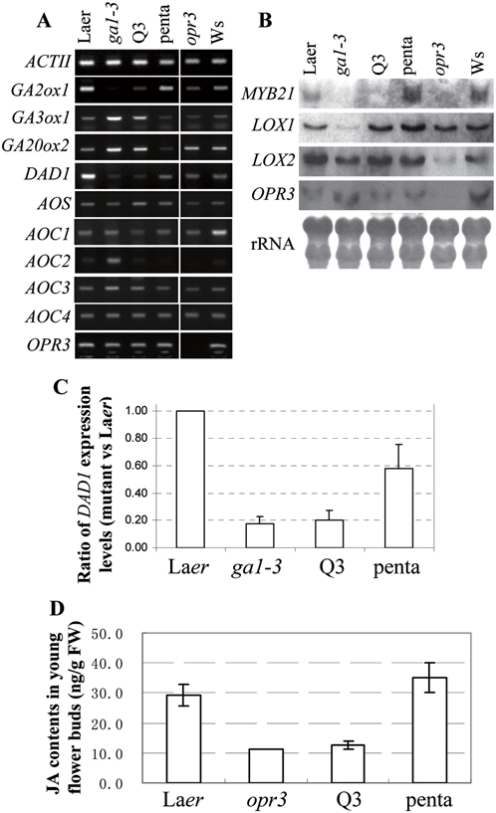
GA Regulates the Expression of JA Biosynthesis Genes *DAD1* and *LOX1*. (A) RT-PCR analysis of GA- and JA-biosynthesis genes in the young flower buds of La-*er* WT control, *ga1-3*, *ga1-3 gai-t6 rga-t2 rgl1-1* (Q3), *ga1-3 gai-t6 rga-t2 rgl1-1rgl2-1* (penta), *opr3* (in WS background) and Ws WT control. (B) Northern analysis of *MYB21*, *GA20ox2*, *LOX1*, *LOX2* and *OPR3* in the young flower buds of the La-*er* WT control, *ga1-3*, Q3, penta, *opr3* and Ws WT control. (C) Semi-quantitative analysis of *DAD1* expression in *ga1-3*, Q3 and penta relative to that in WT (La*er*), respectively. Data were averaged from three independent batches of samples and *ACTII* was used as the normalization control. The expression level of WT is set as 1. (D) Comparison of JA contents among WT (La-*er*), *opr3*, *ga1-3 gai-t6 rga-t2 rgl1-1* (Q3) and *ga1-3 gai-t6 rga-t2 rgl2-1 rgl1-1* (penta) (Table S2). For WT and the penta mutant JA contents were averaged from four repeats. For the Q3 mutant, JA was detected in three out of the four repeats. For opr3, JA was detected only in one out of the four repeats. FW, fresh weight.

We showed in the above that the expression of *MYB21*, *MYB24* and *MYB57* was repressed in the *ga1-3 gai-t6 rga-t2 rgl1-1* quadruple mutant (wild type for *RGL2*) but restored to normal in the *ga1-3 gai-t6 rga-t2 rgl1-1 rgl2-1* penta mutant (Figure 2A and 2B). Mandaokar et al reported that the expression of *MYB21* and *MYB24* was downregulated in *opr3* mutant and application of exogenous JA could restore their expression [32]. These results suggest that there might be a crosstalk between the GA and JA pathways in regulating the expression of *MYB21*, *MYB24* and *MYB57* during stamen development. Genetically, there are three possible ways of interaction between GA and JA. Firstly, GA might act through the JA pathway to regulate the expression of these *MYB* genes. In this case it is expected that JA application onto *ga1-3 gai-t6 rga-t2 rgl1-1* would induce the expression of *MYB21*, *MYB24* and *MYB57* whilst GA application onto *opr3* would have no effect on their expression. Conversely, JA may act upstream of the GA pathway to regulate the expression of these three *MYB* genes. In this case, GA application onto *opr3* would induce whilst JA application onto *ga1-3 gai-t6 rga-t2 rgl1-1* would have no effect on the expression of *MYB21*, *MYB24* and *MYB57*. The third possibility is that GA and JA may not act in a hierarchical manner but rather via parallel pathways to regulate the expression of the three *MYB* genes. If this is the case, GA application onto *opr3* and JA application onto *ga1-3 gai-t6 rga-t2 rgl1-1* would probably both induce the expression of the three *MYB* genes. To find out which is the likely case, we first examined the effect of GA application on JA-deficient mutant *opr3* and found that GA application failed to rescue the *opr3* mutant phenotype and failed to induce the expression of *MYB21*, *MYB24* and *MYB57* in *opr3* even at 96 hrs after GA treatment (Figure 5A). Failure in induction of expression of *MYB21*, *MYB24* and *MYB57* in GA-treated *opr3* mutants could be due to inactivation of GA signaling in JA-deficient background. *GA3ox1* and *GA20ox2* are two key genes that contribute to the biosynthesis of bioactive GA and these two genes are under negative feedback regulation by GA signaling pathway (GA-down) [30]. On the other hand, *GA2ox1* is a GA-up gene responsible for GA catabolism [30]. Examination of the *GA3ox1* and *GA20ox2* and *GA2ox1* expression in GA-treated *opr3* mutants showed expected GA-response (Figure 5B). Meanwhile, expression of *GA3ox1* and *GA2ox1* appeared normal in *opr3* (Figure 6A). These results suggest that JA-deficiency specifically blocks the GA-signaling leading to the induction of *MYB21*, *MYB24* and *MYB57* expression but not the negative feedback pathway for GA-biosynthesis.

### JA Application Restores the Expression of *MYB21*, *MYB24* and *MYB57* in GA-Deficient Mutant

We then studied the effect of JA application on *ga1-3 gai-t6 rga-t2 rgl1-1* (GA-deficient) by examining the expression of *MYB21*, *MYB24* and *MYB57* in the young flower buds at 18, 48, 72 and 96 hrs post-treatment. As expected, *LOX2*, a JA-response gene, was strongly upregulated by JA application at 18 hrs post treatment (Figure 5C) [39]. Interestingly, we observed that JA-treatment induced high expression of *MYB21* and *MYB24* and weak expression of *MYB57* in the *ga1-3 gai-t6 rga-t2 rgl1-1* quadruple mutant at 18 hrs post treatment (Figure 5D). However, examination of *GA3ox1* and *GA20ox2* (two GA-down genes) and *GA2ox1* (GA-up gene) showed that JA treatment did not obviously change the expression patterns of these three GA-response genes in the *ga1-3 gai-t6 rga-t2 rgl1-1* quadruple mutant (Figure 5C). These data suggested that JA signaling might mediate a specific branch of GA signaling to regulate the expression of the three *MYB* genes.

### GA Suppresses DELLA to Upregulate the JA-Biosynthesis Gene *LOX1* and *DAD1*

Considering the fact that JA application was able to induce the expression of *MYBs* in the *ga1-3 gai-t6 rga-t2 rgl1-1* mutant it is reasonable to argue that JA biosynthesis, instead of JA signaling pathway, is likely affected in the *ga1-3 gai-t6 rga-t2 rgl1-1* mutant. To test this hypothesis, we examined the expression of known or putative JA biosynthesis genes including *DAD1* (*Defective in anther dehiscence 1*), *LOX1* (*Lipoxygenase 1*), *LOX2* (*Lipoxygenase 2*), *AOS* (*Allene oxide synthase*), *AOC1* (*Allene oxide cyclase 1*, At3g25760), *AOC 2* (At3g25770), *AOC 3* (At3g25780), *AOC 4* (At1g13280) and *OPR3* (*OPDA reductase 3*) in La-*er* WT, Ws WT, *ga1-3* single, *ga1-3 gai-t6 rga-t2 rgl1-1* quadruple, *ga1-3 gai-t6 rga-t2 rgl1-1 rgl2-1* penta, and *opr3* mutants. We found that only *MYB21*, *LOX2* and *AOC1* showed reduced expression in the *opr3* mutant whereas all the other genes, including GA-biosynthesis genes, expressed similarly in the *opr3* mutant and Ws WT control (Figure 6A and 6B), suggesting that JA-deficiency does not affect GA biosynthesis. Expression of these genes in GA-related mutants was more complicated. We found that the expression levels of *AOS*, *AOC1*, *AOC3*, *AOC4*, *LOX2* and *OPR3* did not show significant differences in all GA-related mutants when compared to the La-*er* WT control (Figure 6A and 6B), suggesting these genes are probably regulated in a GA-independent fashion. The expression of *AOC2* was obviously induced in *ga1-3* and then reduced to the WT level in the quadruple and penta mutants (Figure 6A), suggesting *AOC2* is a GA-down gene. In contrast, the expression of *LOX1* was significantly reduced in *ga1-3* but was restored both in the *ga1-3 gai-t6 rga-t2 rgl1-1* quadruple and penta mutants (Figure 6B), suggesting that although *LOX1* is a GA-up gene and its expression is not repressed by RGL2. Interestingly, *DAD1* expression was found to be downregulated to approximately 20% of the WT level in both *ga1-3* and the *ga1-3 gai-t6 rga-t2 rgl1-1* quadruple mutant whereas was restored to approximately 60% of the WT level in the penta mutant (approximately three folds increase in penta versus Q3) (Figure 6A and 6C), indicating that GA may regulate *DAD1* expression via suppression of RGL2.

### JA Levels Are Greatly Reduced in the Young Flower Buds of the *ga1-3 gai-t6 rga-t2 rgl1-1* Quadruple Mutant

One expected consequence of downregulation of *DAD1* expression by RGL2 is the reduction of JA levels in the *ga1-3 gai-t6 rga-t2 rgl1-1* quadruple mutant (Q3). To text this hypothesis, we measured the JA contents in the young flower buds in WT, *opr3*, the Q3 quadruple mutant and the *ga1-3 gai-t6 rga-t2 rgl1-1 rgl2-1* penta mutant (penta). The data obtained clearly showed that the JA content was greatly reduced in the young flower buds of the quadruple Q3 mutant whereas was restored in the penta mutant when compared to that in the WT and *opr3* mutant (Figure 6D; Table S2).

### GA Application Induces *DAD1* Expression Prior to the Induction of *MYB21*, *MYB24*, and *MYB57*

**Figure 7:**
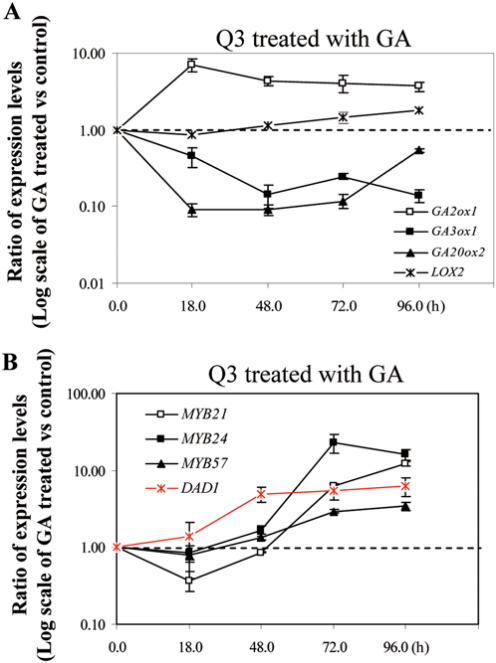
GA Induces *DAD1* Expression Prior To Induction of Expression of *MYB21*, *MYB24*, and *MYB 57*. (A–B) Semi-quantitative analysis of *LOX2*, *GA2ox1*, *GA3ox1* and *GA20ox2* (A), *DAD1* (in red line), *MYB21*, *MYB24* and *MYB57* (B) expression in the *ga1-3 gai-t6 rga-t2 rgl1-1* (Q3) mutant flowers at 18, 48, 72 and 96 hrs after GA treatment. Data were averaged from 2–4 batches of independently treated samples and *ACTII* was used as the normalization control. The graph was drawn based on Log_10_ scale of the ratio of the expression levels of GA treated versus untreated samples.

*DAD1* is a stamen specific gene encoding chloroplastic phospholipase A1 protein that catalyzes the first step of JA biosynthesis. Mutation in *DAD1* resulted in a typical JA-deficient phenotype in stamen development [40], a phenotype similar to that of *myb21-t1 myb24-t1* double mutant. As mentioned earlier, JA likely acts downstream of GA to regulate the expression of *MYB21*, *MYB24* and *MYB57*. To study whether there is a correlation between GA-regulated *DAD1* expression and *MYB21*, *MYB24* and *MYB57* expression, we treated the *ga1-3 gai-t6 rga-t2 rgl1-1* quadruple mutant with GA. We first confirmed the GA-responsiveness in the quadruple mutant plants by examining the expression of known GA-response genes *GA3ox1*, *GA20ox2* and *GA2ox1* (Figure 7A). Then we examined the expression of *DAD1* and the three *MYB* genes *MYB21*, *MYB24* and *MYB57*. Surprisingly, compared to the induction of *MYB21* and *MYB24* expression by JA treatment which is detectable at 18 hrs post treatment (Figure 5D), GA induction of the expression of these two *MYB* genes in *ga1-3 gai-t6 rga-t2 rgl1-1* happens much later and only became detectable at 72 hrs (Figure 7B). More interestingly, GA induction of the expression of *DAD1* is obviously detectable at 48 hrs which is prior to GA-induced expression of *MYB21* and *MYB24* in the *ga1-3 gai-t6 rga-t2 rgl1-1* quadruple mutant (Figure 7B). Our data suggest that GA might first induce the expression of *DAD1* to promoter JA production then via JA signaling to regulate the expression of *MYB21* and *MYB24*.

### Expression of *MYB21*, *MYB24*, and *MYB57* Is Necessary But Insufficient for Normal Stamen Filament Elongation in *ga1-3 gai-t6 rga-t2 rgl1-1*

**Figure 8:**
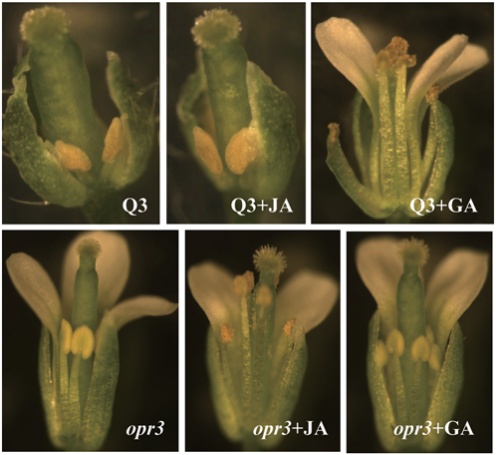
*MYB21*, *MYB24*, and *MYB57* Are Necessary but Insufficient to Complete the Normal Stamen Filament Development. Pictures are shown to compare the stamen phenotype in JA or GA repeatedly treated *ga1-3 gai-t6 rga-t2 rgl1-1* (Q3) and *opr3* plants with respective untreated controls.

As shown in the above, MYB21, MYB24 and MYB57 act downstream of DELLAs in controlling stamen filament elongation. Expression of *MYB21*, *MYB24* and *MYB57* was repressed and floral development was arrested in the *ga1-3 gai-t6 rga-t2 rgl1-1* Q3 quadruple mutant (Figure 2B and 2C). Regarding the fact that JA content is reduced in the young flower buds of Q3 we questioned whether restoration of expression of these *MYBs* by exogenous application of JA could rescue the stamen development to the *ga1-3 gai-t6 rga-t2 rgl1-1* Q3 plants. We analyzed the flowers of JA-treated *ga1-3 gai-t6 rga-t2 rgl1-1* plants and found that repeated JA application was unable to rescue the stamen development (Figure 8) though JA could restore the expression of the three *MYB* genes (Figure 5D), indicating that expression of *MYB21*, *MYB24* and *MYB57* alone was insufficient for normal stamen development in the *ga1-3 gai-t6 rga-t2 rgl1-1* mutant. Furthermore, we found that exogenous application of GA to the *ga1-3 gai-t6 rga-t2 rgl1-1* plants was able to induce the expression of *MYB21*, *MYB24* and *MYB57* (Figure 7B) and recover normal floral development (Figure 8). Taken together, our results demonstrate that besides these JA-inducible *MYBs*, other important GA-regulated JA-independent factors are needed for normal stamen filament development in *ga1-3 gai-t6 rga-t2 rgl1-1*.

### Overexpression of *MYB21* Restored Stamen Filament Elongation and Fertility to *opr3* Flowers

**Figure 9:**
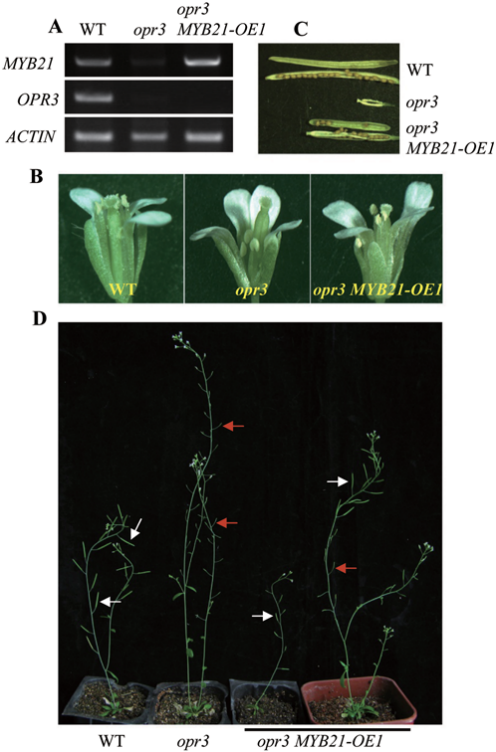
Overexpression of *MYB21* Rescues the Stamen Filament Growth and Fertility to the *opr3* Mutant. (A) RT-PCR analysis of *MYB21* and *OPR3* gene expression in WT, *opr3* and *opr3 MYB21OE-1*. Total RNA was extracted from the young flower buds. *ACTIN* was used as the normalization control. (B) Comparison of the flowers at stage 14 in different genotypes. The flower in *opr3 MYB21OE-1* shows elongated filament than that in *opr3*. (C and D) Comparison of seed set in different genotypes as shown (C) and of plant growth of WT (Col-*0*) (50 days old), *opr3* (50 days old) and *opr3 MYB21OE-1*. The third plant from left was an *opr3 MYB21OE-1* plant with primary shoot (50 days old) whereas the last plant was a 60-day-old *opr3 MYB21OE-1* with axillary shoots after its primary influence has been removed earlier. White arrows highlight siliques with seed set, red arrows highlight sterile siliques.

To test our hypothesis that GA acts through JA to control expression of the *MYB* genes to promote filament elongation, we fused *MYB21* gene with the *CaMV35S* promoter (pCAMBIA1301 vector) and this construct was used to generate transgenic plants in the *opr3* mutant background. Semi-quantitative RT-PCR showed that *MYB21* was overexpressed in the transgenic plants in the *opr3* background (Figure 9A). We found that overexpression of *MYB21* could restore the stamen filament growth (Figure 9B) and restore the fertility (Figure 9C and 9D) to the *opr3* mutant partially. Together with the fact that loss of function of four DELLA (GAI, RGA, RGL1 and RGL2) could not restore the fertility and filament elongation to the *coil1* mutant (Figure S6), we have now provided strong evidence to show that GAs act through JA to control expression of the *MYBs* and promote stamen filament elongation.

## Discussions

In this report, we first identified 34 DELLA-repressed stamen-enriched genes by RT-PCR analysis of candidate genes selected from microarray data [30]. We then selected *MYB21*, *MYB24* and *MYB57* for detailed genetic analysis because GAMYBs are the best characterized transcription factors involved in GA-response. We showed that *Arabidopsis MYB21*, *MYB24* and *MYB57* are highly expressed in the stamen. The stamen-enriched expression pattern is consistent with the observation that the *myb21-t1 myb24-t1 myb57-t1* triple mutant is impaired in the stamen development, especially in the stamen filament elongation. During the course of studying these three *MYBs*, Mandaokar et al reported that *MYB21* and *MYB24* are also JA-inducible [32] which immediately attracted our attention to study the hierarchical relationship between GA and JA in regulating the expression of these three *MYBs*.

We first tested the responses of GA- and JA-deficient mutants (i.e. *ga1-3 gai-t6 rga-t2 rgl1-1* Q3 quadruple mutant and *opr3* mutant, respectively) to GA and JA treatments and found that JA- treatment induced the expression of *MYB21*, *MYB24* and *MYB57* in the GA-deficient plants whereas GA-treatment failed to do so in the JA-deficient plant. This result suggests that JA likely acts downstream of GA pathway to control the expression of these three *MYBs*. It is possible that JA acts downstream by modulating the stability or activity of DELLA proteins to induce the expression of the three *MYBs*. If this is the case, we would expect that JA-treatment would lead to RGL2 degradation or would change the expression patterns of GA-response genes in *ga1-3 gai-t6 rga-t2 rgl1-1*. However, we found that neither the RGL2 protein level (data not shown) nor the expression patterns of three GA-response genes *GA2ox1*, *GA3ox1* and *GA20ox1* were obviously altered in the JA-treated *ga1-3 gai-t6 rga-t2 rgl1-1* plants at 18 hrs post treatment although the three *MYBs* are highly expressed at this time point, suggesting that destabilization or inactivation of DELLA proteins is unlikely the cause for JA-induced expression of *MYBs* in *ga1-3 gai-t6 rga-t2 rgl1-1*. Alternatively, it is possible that GA suppresses DELLA to promote JA production or modulate JA-signaling to induce the expression of the three *MYBs*. The fact that JA application can restore the expression of the three *MYBs* in the GA-deficient background strongly suggests that, at least in part, JA biosynthesis is impaired in the *ga1-3 gai-t6 rga-t2 rgl1-1* mutant.

JA biosynthesis is accomplished by a sequential biochemical reactions mediated by JA-biosynthesis genes including *DAD1*, *LOX1*, *2*, *AOS*, *AOC1*, *2*, *3*, *4* and *OPR3* and is regulated by OPDA compartmentalization and a JA-mediated positive feedback loop [41]. Biotic and abiotic stresses also induce JA formation [42]–[44]. In our experiment, we found that JA biosynthesis gene *DAD1* was greatly down-regulated in both *ga1-3* single and *ga1-3 gai-t6 rga-t2 rgl1-1* quadruple mutants but partially restored to a relatively high level in the penta mutant, suggesting that GA is required for the expression of *DAD1* to control the production of JA via repression of DELLA proteins. In flowers of *dad1* null mutant, the JA levels were only 22% of that of WT [40], demonstrating that limited initial substrate generation by DAD1 reaction acts as a control point for JA biosynthesis in flowers. Therefore, it is highly possible that reduced expression of *DAD1* in *ga1-3 gai-t6 rga-t2 rgl1-1* or *ga1-3* mutant may result in relative low JA production. This hypothesis is strongly supported by the observation that JA content was greatly reduced in the young flower buds of the GA-deficient quadruple mutant *ga1-3 gai-t6 rga-t2 rgl1-1* (Q3 mutant). Furthermore, the fact that the induction of *DAD1* expression happens prior to the expression of *MYBs* by GA in the *ga1-3 gai-t6 rga-t2 rgl1-1* mutant strongly support our hypothesis that GA may regulate the *MYBs*' expression via mobilization of the biosynthesis of JA. A recent report showed that *DAD1* expression is directly controlled by AGAMOUS (AG) [45]. Interestingly, Yu et al reported that *AG* expression was downregulated in the GA-deficient mutant *ga1-3* and exogenous GA application promoted the *AG* expression [20]. It will be interesting to study if there is a relationship among DELLAs, AG and DAD1 in the future. High level of JA would induce the expression of the three *MYB* genes essential for stamen development. In addition to *DAD1*, we also observed that expression of *LOX1* was down-regulated in *ga1-3* mutant and restored to the WT level in the penta mutant. On the other hand, another JA biosynthesis gene *AOC2* was up-regulated in *ga1-3* mutant. These observations suggested that GA may be one of the endogenous signal involved in the regulation of JA biosynthesis genes.

Genetic studies have shown that *MYB21*, *MYB24* and *MYB57* are indispensable for stamen development. The stamen phenotype of *myb21-t1 myb24-t1 myb57-t1* triple mutant is similar to that of JA-deficient mutants including *opr3* and *dad1* mutants. Overexpression of *MYB21* restored the stamen filament elongation and fertility to the *opr3* flowers, strongly suggesting that JA-mediated stamen filament growth is mainly through the MYB pathway. Both *ga1-3* single and *ga1-3 gai-t6 rga-t2 rgl1-1* quadruple mutants showed a more severe flower phenotype than *myb21-t1 myb24-t1 myb57-t1* triple mutant. The fact that expression of these *MYBs* in *ga1-3 gai-t6 rga-t2 rgl1-1* plants was not enough to rescue the mutant flower phenotype indicates that these *MYBs* are necessary but not sufficient for GA-mediated floral development. These data also indicate that modulation of JA pathway may be only one of the branches of GA function in regulating stamen development.

Active cross-talk between different hormone signaling pathways have been revealed in many developmental processes [46]. For example, it was reported that auxin was necessary for GA-mediated *Arabidopsis* root growth by promoting GA-dependent degradation of DELLA proteins [47]. In contrast, ethylene inhibits *Arabidopsis* root growth by delaying the GA-induced destabilization of DELLA [48]. Recently, the complexity of interactions between ethylene and GA signal transduction pathways were analyzed by using combinations of different ethylene and GA related mutants [49]. Hormone-hormone interaction also plays an important role in controlling flowering. For example, it was found that stress induced hormone ethylene control floral transition via DELLA-dependent regulation of floral meristem-identity genes *LEAFY* and *SUPPRESSOR OF OVEREXPRESSION OF CONSTANS 1* (*SOC1*) [50]. We have here established a linear relationship between GA and JA in that GA modulates the expression of *DAD1* that in a likely scenario to promote JA biosynthesis and in return JA induces the expression of *MYB21*, *MYB24* and *MYB57* to control the normal stamen development in *Arabidopsis*.

## Materials and Methods

### Plant Materials

Plants were grown as described previously [19]. Mutant lines (La-*er* background) *ga1-3*, Q3 (*ga1-3 gai-t6 rga-t2 rgl1-1*) and penta (*ga1-3 gai-t6 rga-t2 rgl1-1 rgl2-1*) were described previously [8]. Mutant lines (Col-*0* background) *myb21-t1* (SALK_042711), *myb24-t1* (SALK_017221) and *myb57-t1* (SALK_065776) were obtained from Arabidopsis Biological Resource Centre at the Ohio State University [51] and verified using primer pairs listed in Table 3. These lines were backcrossed twice to purify the genetic background and were then used for all experiments described in this paper. Double mutants were generated from crosses between the relevant single mutants. Triple mutant *myb21-t1 myb24-t1 myb57-t1* was obtained from cross between *myb21-t1 myb24-t1* and *myb24-t1 myb57-t1*. Hexa1 (*ga1-3 gai-t6 rga-t2 rgl1-1 rgl2-1 myb21-t1*), hexa2 (*ga1-3 gai-t6 rga-t2 rgl1-1 rgl2-1 myb24-t1*) and hepta (*ga1-3 gai-t6 rga-t2 rgl1-1 rgl2-1 myb21-t1 myb24-t1*) mutants were in La*er* background via cross-pollination of *myb21-t1 myb24-t1* to the *ga1-3 gai-t6 rga-t2 rgl1-1 rgl2-1* penta mutant four times. SEM of the penta, hexa1, hexa2 and hepta mutants was performed as described previously [8]. The *opr3* mutant is in the Ws background [6].

**Table 3:** Primer pairs used for genotyping MYB mutants.

Mutant lines	Primer pairs used for T-DNA insertion verification	Primer pairs used to amplify fragment spanning T-DNA insertion
*myb21-t1*	LBa1: TGGTTCACGTAGTGGGCCATCG	4334F: ATCGTGCCTATTTCTCCTCCAT
	5355R: TTGATATGATGTCGGTGTAGGAGA	5577R: CGCGGCCGAATAGTTACCATAGT
*myb24-t1*	LBa1: TGGTTCACGTAGTGGGCCATCG	4566F: TGCCGATTCTACCACAAC
	4975R: CTACATCTACGTCGAGCAATAA	4975R: CTACATCTACGTCGAGCAATAA
*myb57-t1*	LBa1: TGGTTCACGTAGTGGGCCATCG	3411F: CATGGTGAAGGTCTTTGGAACT
	3411F: CATGGTGAAGGTCTTTGGAACT	4511R: TAAACAATAACAACGTCCCTTCCT

### Hormone Treatment

Both the *ga1-3 gai-t6 rga-t2 rgl1-1* (Q3) and *opr3* mutant plants (∼27 days old) were sprayed with mock (0.1% ethanol v/v), GA3 (10^−4^ M) (Sigma) or MeJA (0.015% v/v) (Sigma). After treatment, young inflorescences were collected at different time point (18 hrs, 48 hrs, 72 hrs and 96 hrs) for total RNA extraction. For observing rescue of stamen development, mutant plants were repeatedly treated (once a week) with GA or JA.

### RT-PCR and Northern Analysis

Different organs (sepal, petal, stamen, pistil and peduncle) of stage 11–12 flowers were dissected under microscope and pooled for RNA extraction. Flowers younger than stage 11 were pooled as young flower buds for RNA extraction. Total RNA was extracted from the young flower buds of respective genotypes treated with or without GA and JA using Tri Reagent (Molecular Research Center, Cincinnati, OH). The residue genomic DNA in the total RNA was removed via treatment with RNase-free DNase I (Roche, Germany) and the total RNA further purified with the RNeasy Mini kit (QIAgen, Valencia, CA, USA). First strand cDNA was synthesized using SuperScript™II RNase H^−^ Reverse Transcriptase (Invitrogen, USA). First strand cDNA was used as the template in PCR using gene specific primers. Primer pairs used in identification of DELLA-repressed stamen-enriched genes were listed in Table S1. Primer pairs for RT-PCR analysis of *GA2ox1*, *GA3ox1*, *GA20ox2*, *DAD1*, *AOS*, *OPR3*, *LOX1*, *2* and *AOC1*, *2*, *3*, *4* were listed in Table 4. For quantifying the gene expression levels, PCR products were stained with ethidium bromide and the intensity was quantified using software Molecular Analyst (Bio-Rad). The gene expression level was normalized to the expression level of *ACTII* and then displayed as a ratio of expression levels of GA (or JA) treated samples versus untreated control.

**Table 4:** Gene specific primer pairs used in RT-PCR analysis.

Genes	Primers	Genes	Primers
*AtMYB21*	5′ AAAATCGCCAAACATCTTCC 3′	*LOX2*	5′ CCCGGCCGTTTATGGTG 3′
	5′ AATTATAACCCCAAACCTCTACAA 3′		5′ GTCTATTTGCCGCTATTATGTATG 3′
*AtMYB24*	5′ ATGCAAAATGGGGAAATAGGTG 3′	*AOS*	5′ GGCGGGCGGGTCATCAAGT 3′
	5′ AAGATCATCGACGCTCCAATAGTT 3′		5′ TCGCCGGAAAATCTCAATCACAAA 3′
*AtMYB57*	5′ GTGCGGCGAGGGAACATAA 3′	*AOC1*	5′ CACGCCCAAGAAGAAACTCACTC 3′
	5′ TCAGCAATAGAAAAACCAAATAAC 3′		5′ GCTGGCTCCACGTCCTTAGA 3′
*GA2ox1*	5′ CGGTTCGGGTCCACTATTTC 3′	*AOC2*	5′ CTCGGAGATCTCGTACCATTCAC 3′
	5′ ACCTCCCATTTGTCATCACCTG 3′		5′ ACTTATAACTCCGCTAGGCTCCAG 3′
*GA3ox1*	5′ GGCCCCAACATCACCTCAACTACT 3′	*AOC3*	5′ CAATGGCTTCTTCTTCTGCTGCTA 3′
	5′ GGACCCCAAAGGAATGCTACAGA 3′		5′CTTCGAATCTGTCACCGCTCTTTT 3′
*GA20ox2*	5′ CCGGCAGAGAAAGAACACGAA 3′	*AOC4*	5′ TCCCCTTCACAAACAAACTCTACA 3′
	5′ TACGCCTAAACTTAAGCCCAGAA 3′		5′ GGACGGGACACATTACGCTTACG 3′
*DAD1*	5′ GGGCCTACTGGAGCAAATCTAAAC 3′	*OPR3*	5′ ACGGCGGCACAAGGGAACTCTAAC 3′
	5′ GTCTCCTCCACGCGTCTCTGTAT 3′		5′ GGGAACCATCGGGCAACAAAACTC 3′
*LOX1*	5′ GGGCTTGAGGTTTGGTATGCTATT 3′		
	5′ AACGCCTCCAACGCTTCTTTCT 3′		

Northern blot hybridization was performed as described [19]. Fragments of *MYB21* (+294 to +801 nt, the A of the start codon ATG = 1), *GA20ox2* (+28 to +627 nt), *LOX1* (+1903 to +2408 nt), *LOX2* (+1278 to +1714 nt), and *OPR3* (+4 to +439 nt) were labeled using PCR DIG probe synthesis kit (Roche, Germany) and used as probes in Northern blot hybridization.

### Quantification of JA

500 mg young flower buds harvested from different genotypes were frozen in liquid N_2_ and ground to a fine powder with a mortar and pestle. Following addition of 600 µL methanol, homogenates were mixed and kept at 4°C overnight, then centrifuged at 4,800 g for 10 min. The supernatant was transferred to a new 5 mL glass tube and the residue was re-extracted with 200 µL of methanol. 3000 µL ddH_2_O was added to the combined extracts and this solution was applied onto the Sep-pak C_18_ cartridge. The cartridge was washed with 200 µL 20% methanol and 250 µL 30% methanol 300 µL, respectively. Finally, the cartridge was eluted with 300 µL 100% methanol and the eluted solution was collected and used as the samples. Pre-prepared JA solutions (three concentrations were used: 10 ng/mL; 100 ng/mL; 1000 ng/mL) were used as the internal normalization standard. Samples were analyzed by a Thermo TSQ Quantum Ultra LC-MS-MS system. 10 µL of sample was injected onto a Hypersil Gold column (150×2.1, 3 µm). The mobile phase comprised solvent A (0.1% formic acid) and solvent B (methanol) used in a gradient mode [time/concentration of A/concentration of B(min/%/%) for 0/90/10; 1/90/10; 10/10/90; 15/10/90; 16/90/10; 28/90/10]. The machine was run with a spray voltage 4800 v, atomization flow 30 mL/min, auxiliary flow: 2 mL/min, capillary transfer temperature 380°C, lens compensation voltage 77 v, molecular ions m/z 133 (JA), collision energy 15 eV and signal collection interval 15–19 min.

### *CaMV35S::MYB21* Transgenic Plants

For *MYB21* overexpression construct, the Arabidopsis *MYB21* was cloned into an overexpression vector using a primerF: (5′-agctctagaAtggagaaaag aggaggaggaag-3′) and a primerR: (5′-atcgagctctcaattaccattcaataaatgca-3′) through *XbaI* and *SacI* sites. The overexpression vector, which was derived from pCAMBIA1301, contains the *CaMV35S* promotor to drive the expression of *MYB21*. The plasmids was confirmed by sequencing and introduced into *Agrobacterium tumefaciens* by electroporation and then introduced into heterzygous *OPR3/opr3* plant by flower dip method [19]. More than 20 transformed lines were obtained based on PCR analysis. Homozygous *opr3* mutants were identified using Opr3-RP (5′-ctcaaatattggcgagacctg-3′) and Opr3-LP (5′-GGCAGAGTATTATGCTCAACG-3′).

### *pMYB24::GUS* Transgenic Plants

To make the *pMYB24::GUS* construct, a 3098 bp (68 bp upstream of *MYB24* start codon ATG) genomic DNA fragment was PCR amplified from Col-0 genomic DNA using primers 18F (*Pst*I, 5′ TTCTAGG**C**TGCAGCTAAACGACTTC 3′) and 2934R (5′ GTAATAGAAAGGGAGAGTTGTGAAAG 3′). PCR amplifications of promoter regions were performed using *PfuTurbo* DNA polymerase (Stratagene). The amplified DNA fragment was digested with *Pst*I and then cloned into *Pst*I/*Nco*I-cleaved pCambia 1301 vector and their sequences were confirmed by sequencing. The *pMYB24::GUS* fusion construct was then introduced into *Arabidopsis thaliana* ecotype Col-*0* plants using flower dip method [19]. More than three independent lines were examined at various stages of floral development in this study.

### *In Situ* Hybridization

Whole inflorescences which included unopened flower buds were fixed and *in situ* hybridization was carried out as described before [8]. Antisense and sense probes of *MYB21* (+294 to +801 nt, nt stands for nucleotides, the A of the start codon ATG = 1) for in situ hybridization were DIG-labeled by *in vitro* transcription.

## Supporting Information

Figure S1 Phylogenetic Tree Showing the Relationship among *MYB21*, *MYB24*, and *MYB57* and Other *MYBs*.
(9.39 MB TIF)

Figure S2 *MYB21* Expression Patterns. (A) Cross section of an anther showing that *MYB21* is expressed in the vascular tissue. VT, vascular tissue. (B) Transverse section of a stamen showing that *MYB21* is expressed in the region linking stamen filament and the anther where fast cell elongation occurs.
(6.07 MB TIF)

Figure S3 *MYB24* Expression Patterns. (A) GUS staining of a young inflorescence from a *pMYB24::GUS* plant. (B–E) GUS staining of flowers at various stages after floral stage 11. GUS activity is clearly detectable after the floral stage 12 (C). Flowers were sequentially taken from the same inflorescence.
(10.33 MB TIF)

Figure S4 Analysis of Stamen and Pistil Length in Different *MYB* Mutants. (A) Ratio of length of stamen to its respective pistil in flowers at the floral stage 12. (B) Number of flowers used in the analysis in (A).
(13.51 MB TIF)

Figure S5 *myb21-t1 myb24-t1* Is Epistatic To *ga1-3 gai-t6 rga-t2 rgl1-1 rgl2-1*. Pictures showing whole inflorescences from hepta (*myb21-t1 myb24-t1 ga1-3 gai-t6 rga-t2 rgl1-1 rgl2-1*), hexa1 (*myb21-t1 ga1-3 gai-t6 rga-t2 rgl1-1 rgl2-1*) and hexa2 (*myb24-t1 ga1-3 gai-t6 rga-t2 rgl1-1 rgl2-1*).
(5.91 MB TIF)

Figure S6 *coi1* Mutation Is Epistatic To *ga1-3 gai-t6 rga-t2 rgl1-1 rgl2-1* (penta) in Stamen Filament Elongation. Flowers from different genotypes at the floral stage 14 were compared. (A) La-*er* WT; (B) penta mutant; (C) *coi1* mutant; (D) *coi1* penta mutant.
(6.83 MB TIF)

Table S1 List of Primers Used in Identifying Stamen-Enriched Genes.
(0.06 MB DOC)

Table S2 JA Contents in Young Flower Buds.
(0.03 MB DOC)
