# Supplementary figures and images for: Gibberellin Acts through Jasmonate to Control the Expression of MYB21, MYB24, and MYB57 to Promote Stamen Filament Growth in Arabidopsis
Source: PLoS Genet. 2009 Mar 27;5(3):e1000440. doi: 10.1371/journal.pgen.1000440 (PMC2654962; doi:10.1371/journal.pgen.1000440)

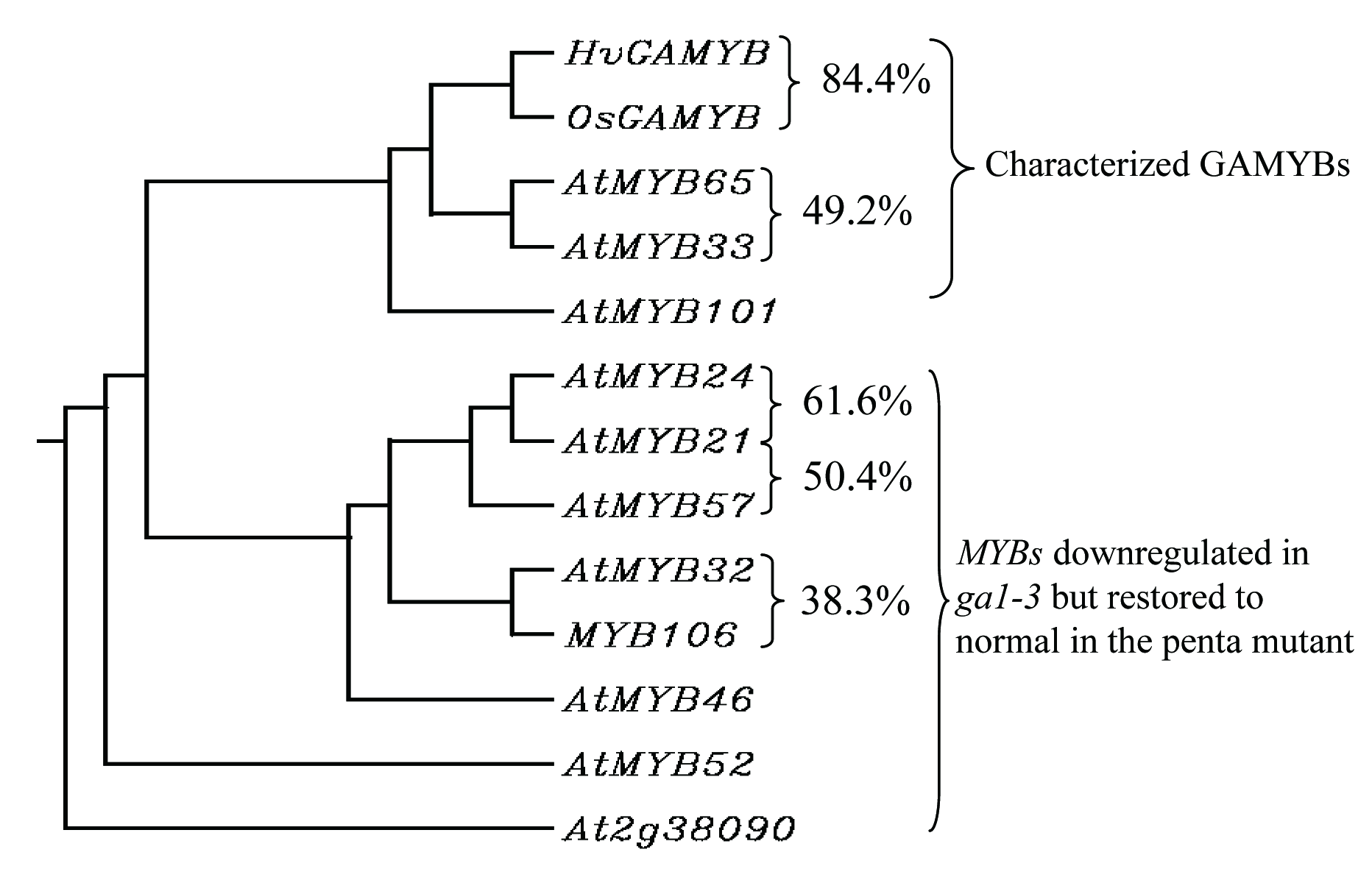

Supplement: Figure S1 — Phylogenetic Tree Showing the Relationship among MYB21, MYB24, and MYB57 and Other MYBs. (9.39 MB TIF) [file pgen.1000440.s001.tif]

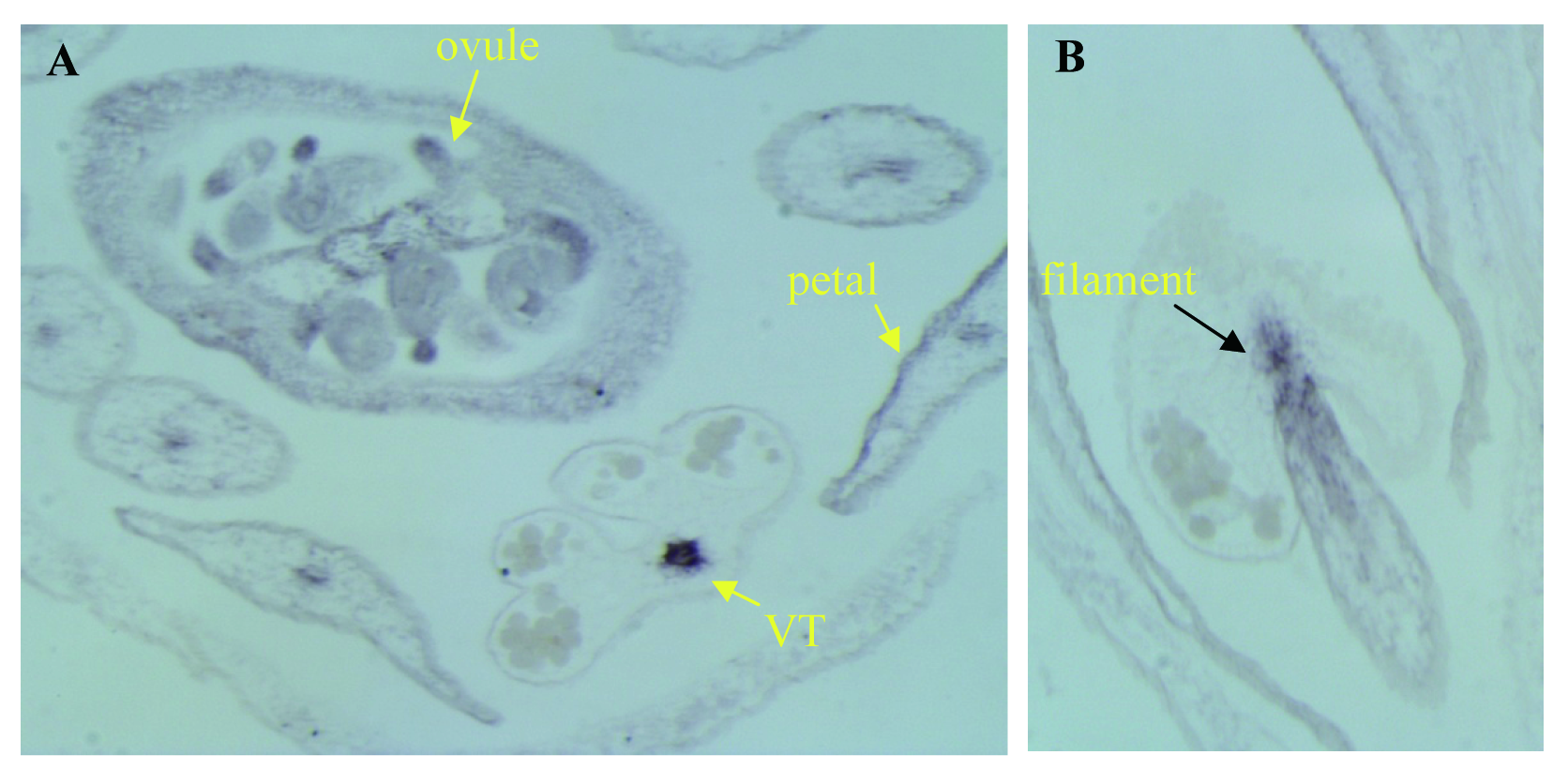

Supplement: Figure S2 — MYB21 Expression Patterns. (A) Cross section of an anther showing that MYB21 is expressed in the vascular tissue. VT, vascular tissue. (B) Transverse section of a stamen showing that MYB21 is expressed in the region linking stamen filament and the anther where fast cell elongation occurs. (6.07 MB TIF) [file pgen.1000440.s002.tif]

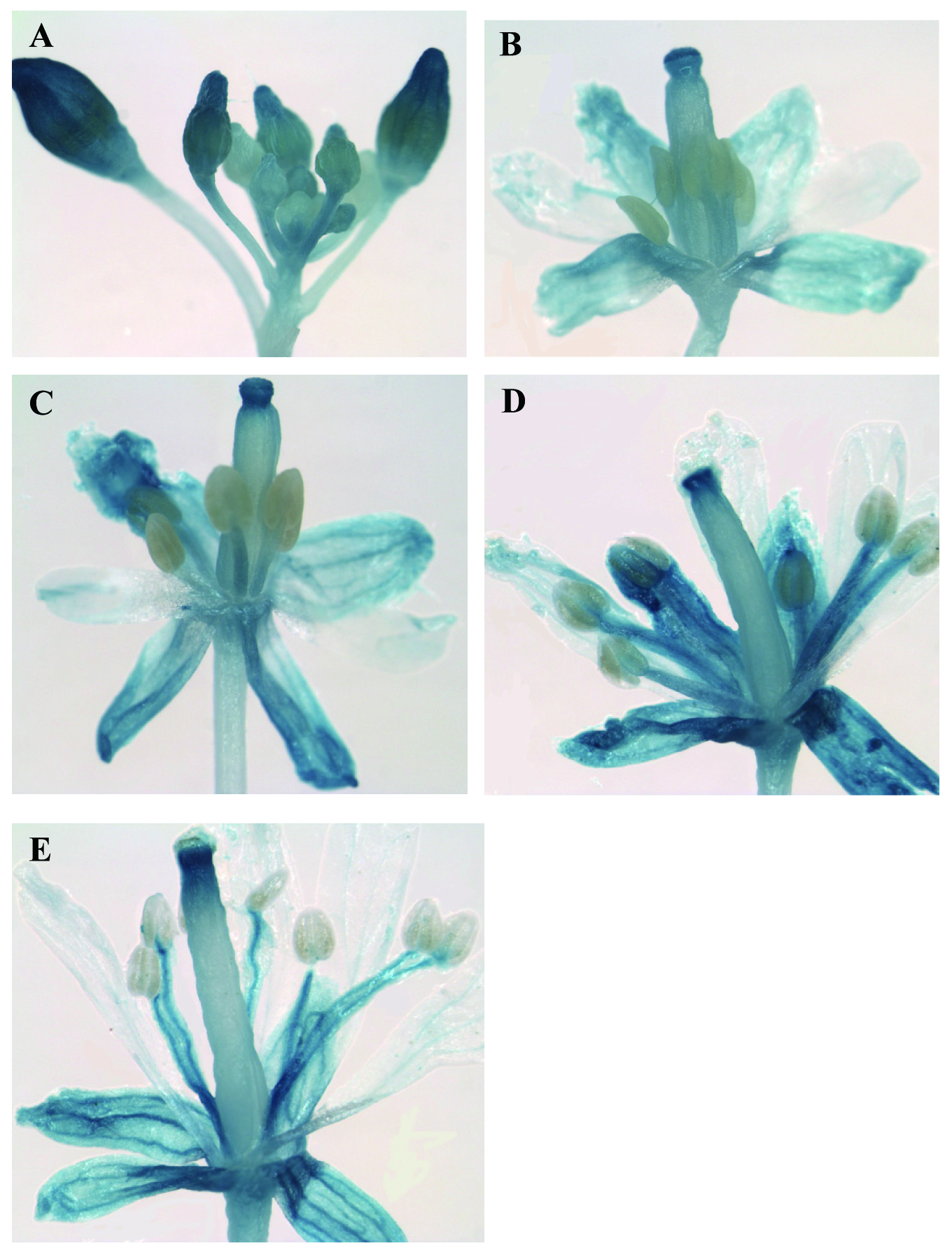

Supplement: Figure S3 — MYB24 Expression Patterns. (A) GUS staining of a young inflorescence from a pMYB24::GUS plant. (B–E) GUS staining of flowers at various stages after floral stage 11. GUS activity is clearly detectable after the floral stage 12 (C). Flowers were sequentially taken from the same inflorescence. (10.33 MB TIF) [file pgen.1000440.s003.tif]

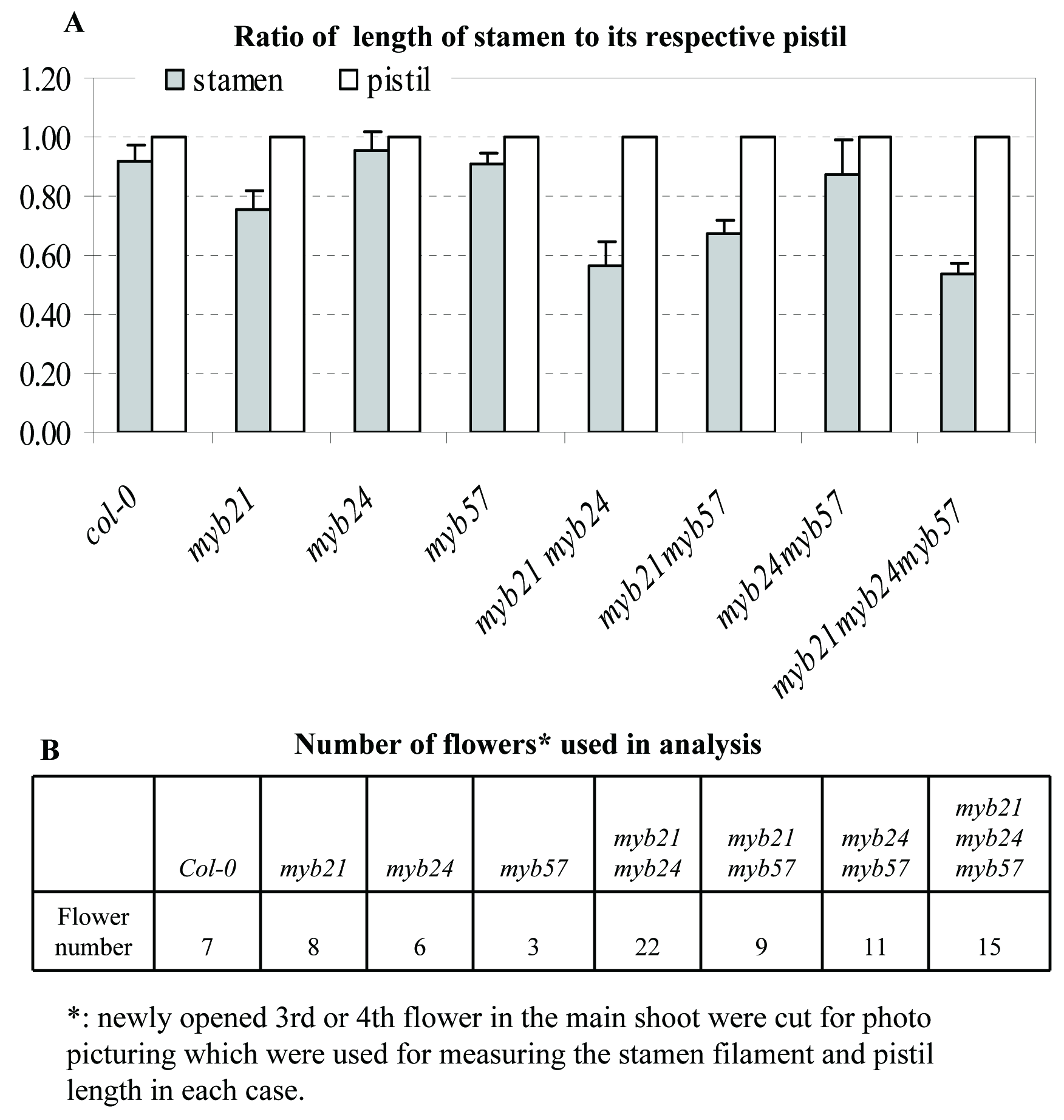

Supplement: Figure S4 — Analysis of Stamen and Pistil Length in Different MYB Mutants. (A) Ratio of length of stamen to its respective pistil in flowers at the floral stage 12. (B) Number of flowers used in the analysis in (A). (13.51 MB TIF) [file pgen.1000440.s004.tif]

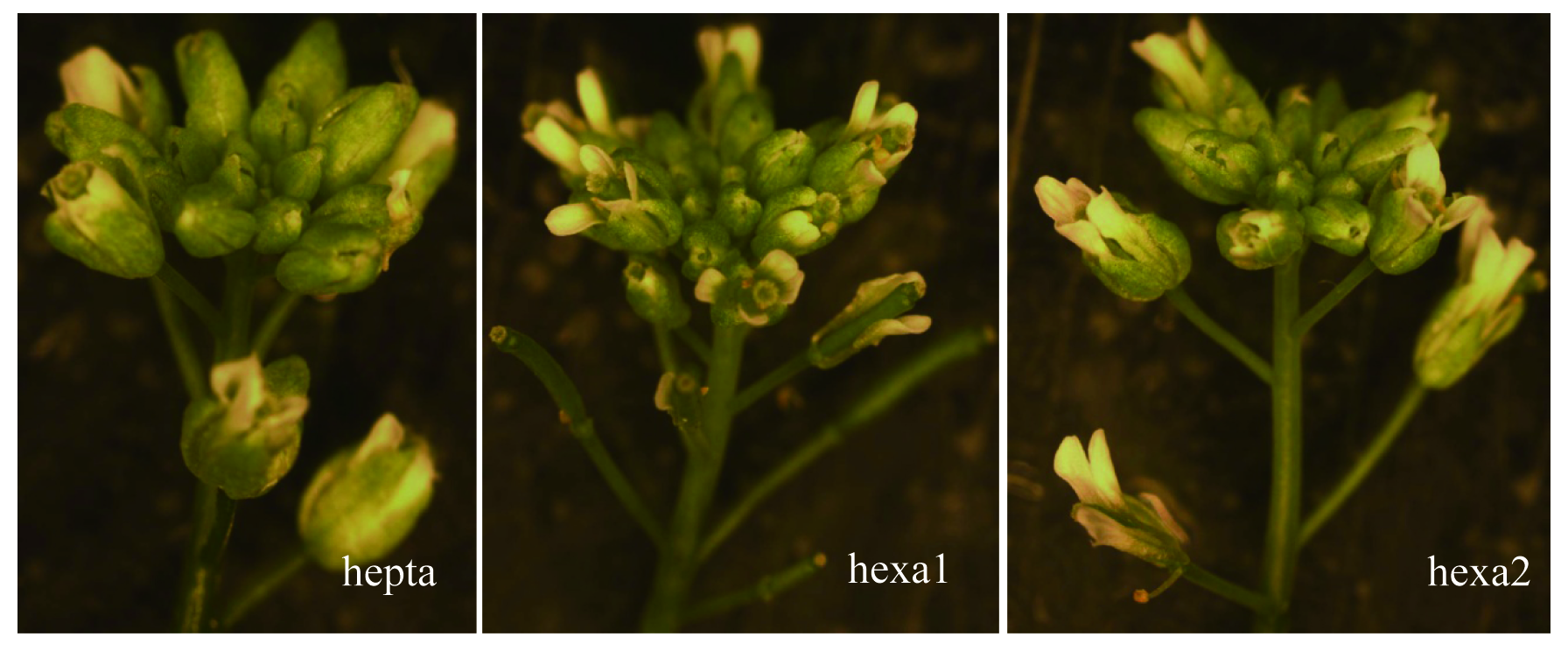

Supplement: Figure S5 — myb21-t1 myb24-t1 Is Epistatic To ga1-3 gai-t6 rga-t2 rgl1-1 rgl2-1. Pictures showing whole inflorescences from hepta (myb21-t1 myb24-t1 ga1-3 gai-t6 rga-t2 rgl1-1 rgl2-1), hexa1 (myb21-t1 ga1-3 gai-t6 rga-t2 rgl1-1 rgl2-1) and hexa2 (myb24-t1 ga1-3 gai-t6 rga-t2 rgl1-1 rgl2-1). (5.91 MB TIF) [file pgen.1000440.s005.tif]

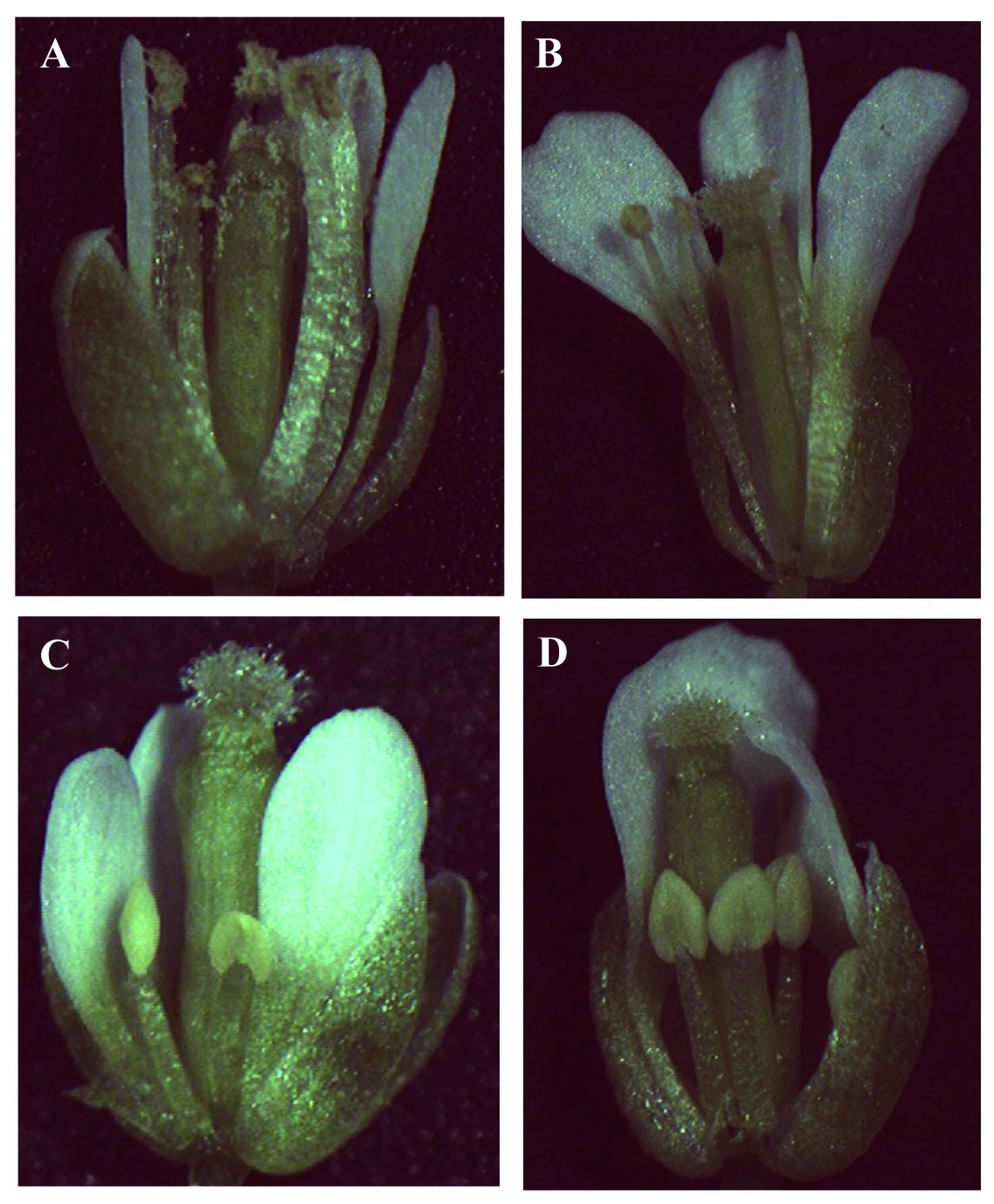

Supplement: Figure S6 — coi1 Mutation Is Epistatic To ga1-3 gai-t6 rga-t2 rgl1-1 rgl2-1 (penta) in Stamen Filament Elongation. Flowers from different genotypes at the floral stage 14 were compared. (A) La-er WT; (B) penta mutant; (C) coi1 mutant; (D) coi1 penta mutant. (6.83 MB TIF) [file pgen.1000440.s006.tif]
