# Supplementary material for: Gibberellin Acts through Jasmonate to Control the Expression of MYB21, MYB24, and MYB57 to Promote Stamen Filament Growth in Arabidopsis
Source: PLoS Genet. 2009 Mar 27;5(3):e1000440. doi: 10.1371/journal.pgen.1000440 (PMC2654962; doi:10.1371/journal.pgen.1000440)
Supplement: Table S1 — List of Primers Used in Identifying Stamen-Enriched Genes. (0.06 MB DOC) [file pgen.1000440.s007.doc]

| **Table S1. List of primers used in identifying stamen-enriched genes.** | | | |
| --- | --- | --- | --- |
| **Gene ID** | **Primer Pairs** | **Gene ID** | **Primer Pairs** |
| At1g09610 | 5’ TACCACGTTCCCTCGCACAAG 3’ 5’ GATCCCAACCGGTCTCATAAAT 3’ | At3g18660 | 5’ TTTGGATTGGCGATGAAGATGACG 3’ 5’ CAAGTTATGGCCGGGAAGTGATGA 3’ |
| At1g17950 | 5’ GTGGCTGCGACTGGGATGATT 3’  5’ TTCTCGTTAGGAATTCGGTTG 3’ | At3g20520 | 5’ TTCCCGGGCTGTACTGACTTG 3’ 5’ GTACCGCATCGACCACGCTAAT 3’ |
| At1g52690 | 5’ AGCCCAGTCAGCCCAACAA 3’ 5’ CGAACGCAACAAACACTAATCAAA 3’ | At3g22800 | 5’ TATCGCCGGTTATCTCCCTTTAG 3’ 5’ ACCGGTTAGTTGTGAGTTCGTGAT 3’ |
| At1g70690 | 5’ GCGGCGACCTAGACCCAACC 3’ 5’ GCGGCTAACATTTCGACAAGTAA 3’ | At3g27810 | 5’ AAAATCGCCAAACATCTTCC 3’  5’ AATTATAACCCCAAACCTCTACAA 3’ |
| At1g75880 | 5’ CGTTCCGGCGGTAATAGTG 3’ 5’ GGGGGAGAGTAAAGAAGTCGTT 3’ | At3g54770 | 5’ GGTGGCCGTCTTCGTAAATC 3’ 5’ TATGGCCTTCTTCTGTATGCTCTC 3’ |
| At1g75900 | 5’ CAGCGACGACATAGCCAATACATA 3’ 5’ AGACACGTCGGGACATACAGAAGA 3’ | At3g62020 | 5’ TCGCACTTCAGGGATAAAA 3’ 5’ CCGGAGTGGCTGTGAATA 3’ |
| At1g76240 | 5’ AGCCGAAGACAAAACACCAACACT 3’ 5’ AACGCAAATAGACAGGAAACAAGA 3’ | At4g09960 | 5’ ACTCGTGGCCGTCTCTATGA 3’ 5’ AGTTATTGCAGCTCGGTTTTTC 3’ |
| At1g78440 | 5’ CGGTTCGGGTCCACTATTTC 3’ 5’ ACCTCCCATTTGTCATCACCTG 3’ | At4g12730 | 5’ CTCCGGCGAACAAGACTGC 3’ 5’ CGGCGCCGTTTTTATCGT 3’ |
| At2g17950 | 5’ ACCATCTTCATCACCCAACTCG 3’ 5’ ATAAGCATCGCCACCACATTCT 3’ | At4g12960 | 5’ CCGATCAGAAATCACAATACTCGTT 3’ 5’ AAGCTTCCATTTCCTCTCAG 3’ |
| At2g34790 | 5' TTCACCGGCCAGTAAAACCACCAA 3' 5’ TTTCCTCGCTCACTTTCCCATCTT 3’ | At4g18780 | 5’ CCGCCTCAAGTTGCTCCAGA 3’  5’ ATCACCCAAAAGGCGAAAAATACC 3’ |
| At2g34810 | 5’ TTCAAGCACCAATCTCAAAACAGG 3’ 5’ AGAACCGGAATCCCTTGCTGAG 3’ | At4g34990 | 5’ GGTTGTTGGCGTTCTCTTCCTA 3’  5’ CACGCACTGCACCTATACTTCACT 3’ |
| At2g34870 | 5’ AACCGTCGCTCGTCGTCTTACTG 3’ 5’ ATCGATCGGTCCTTTATTTCTTAG 3’ | At5g12870 | 5’ AACCCTTCCTTGACCCACATA 3’  5’ CTCAAGCCCTAGTACGAAAAGATT 3’ |
| At2g38080 | 5’ CTACAGCGGAGGATCAGTCACGAA 3’ 5’ ATCCTTAGGCGGCGGCAAAAT 3’ | At5g17420 | 5’ TTGGGCGCCGGAGAAAGA 3’  5’ GAGGACTGTGCCGGCTGAAAAAT 3’ |
| At3g01530 | 5’ GTGCGGCGAGGGAACATAA 3’  5’ TCAGCAATAGAAAAACCAAATAAC 3’ | At5g40350 | 5’ CCAGAAATACATCATCAAGAGCGG 3’  5’ GCCAAAGATCATCGACGCTCC 3’ |
| At3g11480 | 5' AGGAGCTCATGATCACAAACAAAT 3'  5’ CACTCACAAGACCCTCAAAGACTA 3’ | At5g44030 | 5’ CGGAGGCGACGAACACGA 3’  5’ AGAAGACAAACGGCCGGAATAGTA 3’ |
| At3g12000 | 5’ TGGTCGGGATCTCAAAACAGG 3’ 5’ AAGCCCAAGCCGTCACATTCT 3’ | At5g44630 | 5’ AAGCCTGGCCGATTGTTT 3’ 5’ TTCTCTACTGGCTTCTTCTTTTGT 3’ |
| At3g16920 | 5’ CCGTCGGCTTTTGGGATTAC 3’ 5’ TGCTTCTTCGGTGGTGACAT 3’ | At5g59120 | 5’ AGAATCCGCAGGGCTTTGTGAG 3’ 5’ GAT GCGATGCCAGTTCCAGTAGCA 3’ |
