# Supplementary material for: Gibberellin Acts through Jasmonate to Control the Expression of MYB21, MYB24, and MYB57 to Promote Stamen Filament Growth in Arabidopsis
Source: PLoS Genet. 2009 Mar 27;5(3):e1000440. doi: 10.1371/journal.pgen.1000440 (PMC2654962; doi:10.1371/journal.pgen.1000440)
Supplement: Table S2 — JA Contents in Young Flower Buds. (0.03 MB DOC) [file pgen.1000440.s008.doc]

| **Table S2. JA contents in young flower buds.** | | | | |
| --- | --- | --- | --- | --- |
|  | Laer | opr3 | Q3 | penta |
| JA content in young flower buds  （ng/g FW） | 36.515 |  | 13.3485 | 30.55 |
|  | 25.6515 |  |  | 23.7205 |
|  | 34.508 |  | 14.7305 | 44.741 |
|  | 20.399 | 11.2965 | 10.054 | 41.34 |
| Average  （ng/g FW） | 29.3±3.8  (n=4) | 11.3  (n=1) | 12.7±1.4  (n=3) | 35.1±4.8  (n=4) |
